# The performance of protected-area expansions in representing tropical Andean species: past trends and climate change prospects

**DOI:** 10.1038/s41598-022-27365-7

**Published:** 2023-01-18

**Authors:** Javier Fajardo, Janeth Lessmann, Christian Devenish, Elisa Bonaccorso, Ángel M. Felicísimo, Fernando J. M. Rojas-Runjaic, Haidy Rojas, Miguel Lentino, Jesús Muñoz, Rubén G. Mateo

**Affiliations:** 1 Real Jardín Botánico (RJB), CSIC, Plaza de Murillo 2, 28014 Madrid, Spain; 2 Centro Universitario de Mérida, Universidad de Extremadura, Santa Teresa de Jornet 38, 06800 Mérida, Spain; 3 Universidad Internacional Menéndez Pelayo, Madrid, Spain; 4 UN Environment Programme World Conservation Monitoring Centre (UNEP-WCMC), 219 Huntingdon Rd, Cambridge, CB3 0DL UK; 5 Departamento de Ecología, Pontificia Universidad Católica de Chile, Alameda 340, 8331150 Santiago, Chile; 6 Instituto de Ecología y Biodiversidad, Casilla 653, Santiago, Chile; 7 NatureMetrics, 1 Occam Court, Surrey Research Park, Guildford, GU2 7HJ UK; 8 Manchester Metropolitan University, Chester Street, Manchester, M1 5GD UK; 9 Universidad San Francisco de Quito, Instituto Biósfera y Colegio de Ciencias Biológicas y Ambientales, Quito, Ecuador; 10 Museu Paraense Emílio Goeldi, Belém, Brazil; 11 Museo de Historia Natural la Salle, Fundación la Salle de Ciencias Naturales, Caracas, 1050 Venezuela; 12 Laboratorio de Biología de Organismos, Centro de Ecología, Instituto Venezolano de Investigaciones Científicas, 20632, Caracas, 1020 Venezuela; 13 Colección Ornitológica Phelps, Bello Monte Caracas 1060, Caracas, Distrito Capital Venezuela; 14 Departamento de Biología, Facultad de Ciencias, Universidad Autónoma de Madrid, Campus de Cantoblanco, C/Darwin 2, 28049 Madrid, Spain; 15 Centro de Investigación en Biodiversidad y Cambio Global, Universidad Autónoma de Madrid, 28049 Madrid, Spain

**Keywords:** Climate-change ecology, Conservation biology, Biodiversity

## Abstract

Protected area (PA) extent has increased significantly over the last 150 years globally, but it is yet unclear whether progress in expanding coverage has been accompanied by improved performance in ecological representation. Here, we explore temporal trends in the performance of PA networks in representing > 16,000 vertebrate and plant species in tropical Andean countries based on species bioclimatic niche modelling. We use a randomization analysis to assess whether representation gains over time (1937–2015) are the expected consequence of increasing the overall area of the network or the result of better designed networks. We also explore the impact of climate change on protected-area representation based on projected species distributions in 2070. We found that PAs added in the last three to four decades were better at representing species diversity than random additions overall. Threatened species, amphibians and reptiles are the exception. Species representation is projected to decrease across PAs under climate change, although PA expansions over the last decade (2006–2015) better represented species' future bioclimatic niches than did sites selected at random for most evaluated groups. These findings indicate an unbalanced representation across taxa, and raises concern over under-represented groups, including threatened species, and species’ representation under climate change scenarios. However, they also suggest that decisions related to locating protected areas have become more strategic in recent decades and illustrate that indicators tracking representativeness of networks are crucial in PA monitoring frameworks.

## Introduction

Protected area (PA) networks aim to represent biodiversity and enable its persistence in the face of anthropogenic threats, including climate change^1,2^. Well-designed and effectively managed PAs have been able to deliver positive outcomes in terms of limiting the expansion of local stressors^3^, reducing deforestation^4^, or maintaining local biodiversity^5^, among other benefits for nature and people^6,7^. PAs are often considered the keystone of biodiversity conservation, as recognized by the global conservation community and by the Convention on Biological Diversity (CBD), which has engaged nations towards expanding the global PA network^8^. Accordingly, the last decades have seen a pronounced expansion of PAs worldwide, which currently cover 15.8% of the terrestrial surface of the planet^9^. But the steady increase in the total global area protected contrasts with assessments that suggest growing rates of biodiversity loss^10,11^. The discrepancy between both trends supports the idea that indicators other than PA coverage are crucially needed to measure effectively the performance of PA networks, enabling biodiversity conservation^12,13^.

Ecological representation, or PA representativeness, is an indicator of PA performance. It is defined as the ability of PAs to include all biodiversity features of interest (e.g., species, ecosystems)^14^. The concept of representation is often coupled with that of adequacy of PA representation, which specifies that sufficient samples of features (or representation targets) must be included to ensure conservation outcomes^14^. PAs have historically been created within landscapes of low opportunity costs and marginal contribution to enhancing ecological representation^1,15^. These biases in past PA designations have led to suboptimal representativeness of PA in current networks and persisting systemic network deficits in biodiversity coverage^16^. With the aim of reversing this trend, guidance for expanding global PA coverage has highlighted the need to create ecologically representative systems (e.g.,^8,13,16,17^) by strategically locating new PAs in places that contribute the most to closing multiple ecological representation gaps^10,18^.

Despite this knowledge, it is yet unclear whether increasing awareness of the need to enhance the ecological representativeness of conservation networks has translated into improvements in the efficiency with which expansions in PA coverage have contributed to enhancing representation through time. Kuempel et al.^19^ found that protection equality of terrestrial ecoregions, a metric of PA representativeness, improved slowly with PA expansions made through time. Nonetheless, this expansion was inefficient since the ecological representation improved no more than if the same amount of protected land had been established at random. Similarly, Venter et al.^18^ found that recent global PA expansions were highly suboptimal at representing threatened vertebrate species compared to what could have been achieved at the same cost by optimized strategic planning. Interestingly, a regional analysis for Australia showed that PAs designated since the year 2000 were also suboptimal at representing threatened species but performed better than randomly selected areas, suggesting a role of conservation planning in enhancing representation^20^. In light of these mixed results, further research on the performance of past PA expansions with respect to ecological representation is needed in order to understand the success of regional conservation decisions and global biodiversity conventions. Crucially, these assessments should be extended to broader groups of taxa and regions (i.e., not only threatened species), especially in other highly biodiverse areas whose conservation strategies are critical to achieving global biodiversity targets. A temporal perspective in this analysis is also relevant to detect potential improvements in conservation planning decisions over time^20^.

PA representativeness must also be evaluated under climate change scenarios where species are expected to shift their distributions following suitable climate^21^. These shifts are expected to change species composition within PAs, potentially leading to decreases in representation^21–23^. Therefore, it is also relevant to determine whether most recent decisions on designating PAs have also been beneficial when accounting for climate change impacts in ecological representation, or if such adaptability remains to be gained in the future to ensure resilient conservation networks.

**Figure 1:**
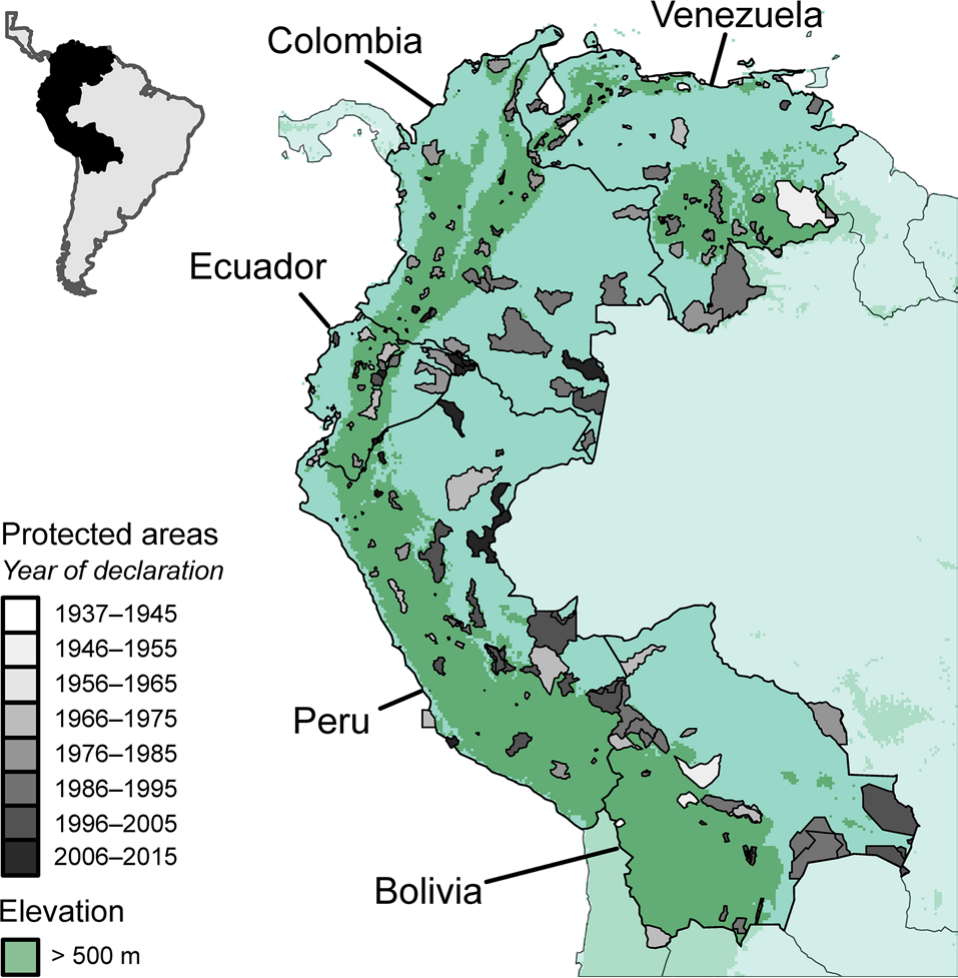
Study area: protected areas in the five tropical Andean countries in South America. Protected area colours indicate their year of declaration from older (light grey) to newer (dark grey). Figure created in R v4.0.0 (https://cran.r-project.org/).

**Figure 2:**
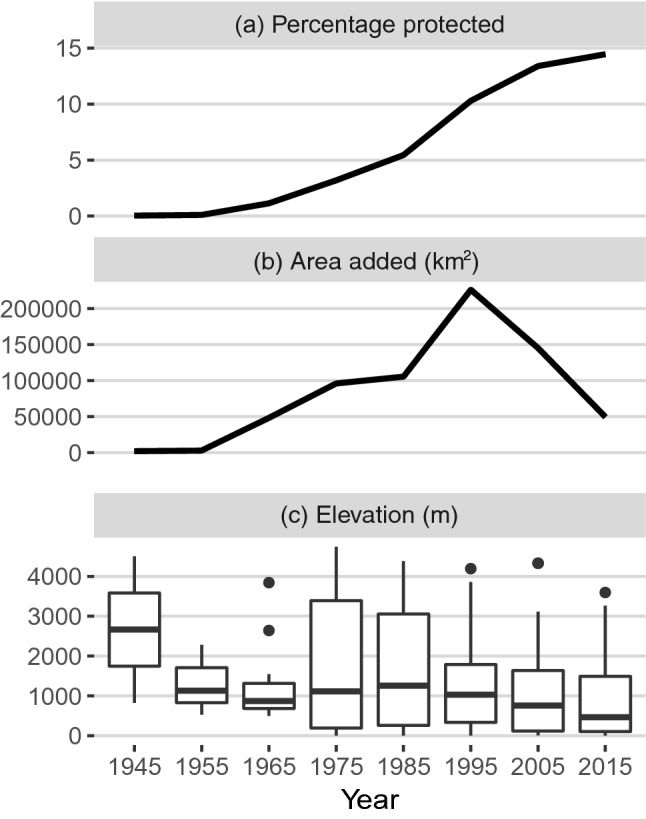
Temporal trends in protected area coverage in the Tropical Andes. (**a**) Percentage of the countries’ terrestrial area included in the conservation network. (**b**) Amount of area added to the network per decade. (**c**) Average elevation of protected areas declared per decade. Figure created in R v4.0.0 (https://cran.r-project.org/).

Here, we explore temporal trends in the performance of PAs in representing > 16,000 vertebrate and plant species in the tropical Andean countries (Venezuela, Colombia, Ecuador, Peru, and Bolivia, Fig. 1). PA networks in tropical Andean countries have expanded significantly since their first PA was declared in 1937 (Fig. 2), but there is concern over the ability of these networks to adequately represent biodiversity and maintain its properties under climate change^24,25^. Using species distribution models to illustrate current biodiversity patterns and to project distribution shifts under a 2070 climate change scenario, in addition to using a null model to compare the performance of PAs against randomized networks, we evaluate the PA networks in tropical Andean countries, with regard to: (1) temporal trends in species representation (1937–2015), accounting for adequacy of representation targets and differences among taxa and threat status; (2) the estimated representation of taxonomic groups/threatened species in the 2015 network under climate-change scenarios; and (3) whether representation gains over time were just the expected consequence of increasing the overall area of the network or the result of selecting more efficient sites for the PAs that were added to the network. Tropical Andean countries hold the responsibility to preserve a considerable portion of the world’s biodiversity because they include one of the richest regions on Earth in terms of species diversity and endemism^26^, making their conservation both regionally and globally relevant.

## Results

### Species representation over time

**Figure 3:**
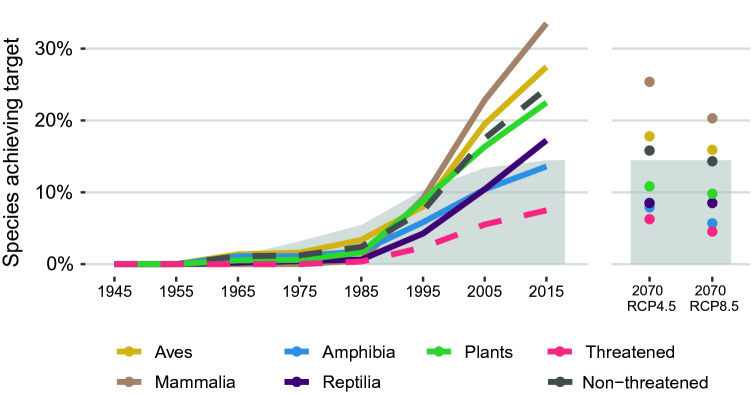
Temporal trends of species representation in expanding protected-area networks by taxonomic groups (colours) and threat condition of all groups (dotted line). On the right, representation in the two projected climate-change scenarios of intermediate (RCP4.5) and high (RCP8.5) emissions. The percentage of the country area protected is shown by grey shading. Figure created in R v4.0.0 (https://cran.r-project.org/).

We assessed trends in ecological representation across the combined PA network of the five tropical Andean countries by assessing achievement of representation targets (i.e., the percentage of distributions under protection in order for species to be considered as represented)^1,27^ for 16,510 species of vertebrates and plants. The analysis was based on the modeled distributions of 1810 birds, 197 mammals, 699 amphibians, 452 reptiles, and 13,352 plants built with ensembles of three modeling techniques^28–31^. Evaluating the PA network on a decadal basis since the first reserve was declared in 1947 in Venezuela until 2015, we found that only a small proportion of species (~ 2%) had met their conservation targets by 1985 (Fig. 3) despite the total area of PA networks already consisting of half the area by 2015. The decade 1986–1995, corresponding to the largest enlargement, triggered a marked increase in species representation, but with mammals, birds, and plants boosting their representation at higher rates than reptiles and amphibians. Today, mammals and birds are the best represented groups, with 33.5% and 27.5% of their species meeting targets, respectively. Representation is smaller for plants (22.6%), reptiles (17.2%), and amphibians (13.6%).

Crucially, threatened species^32^ were, on average, less represented (8.4%) than non-threatened (24.4%) in the 2015 PAs, resulting from a trend in which decadal increases in representation were on average comparatively less beneficial for these species with higher conservation needs.

### Change in performance in species representation

**Figure 4:**
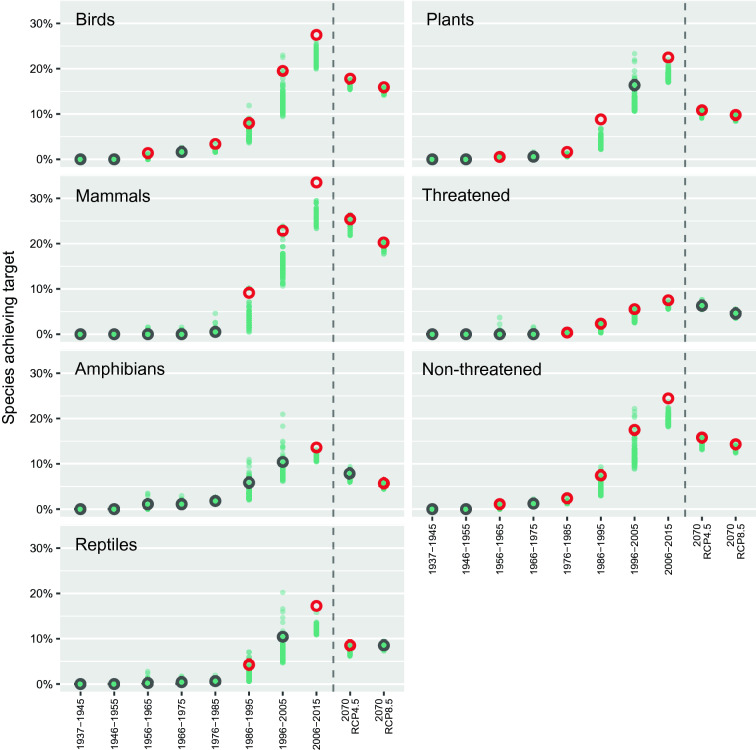
Decadal change in species representation in PAs showing, for each decadal expansion of the network, the compared performance of the actual PAs against randomly simulated expansions. Results are shown for birds, mammals, amphibians, reptiles, plants, all threatened taxa, and all non-threatened taxa. Representativeness achieved by the actual PA network is represented by open points, and that of each of the 100 random networks by smaller green points. An open red point indicates that the representativeness of the real PAs is higher than expected by chance. The two rightmost points correspond to representativeness in the two climate-change scenarios of intermediate (RCP4.5) and high (RCP8.5) emissions, considering no expansion of the 2015 network. Figure created in R v4.0.0 (https://cran.r-project.org/).

Using a randomization analysis^33^ to test whether the performance of decadal additions to the regional PA network contributed more than randomly placed PAs to enhance species representation, we observed an overall trend of increasing performance with successive PA network expansions. Whereas typically PAs declared during the first decades did not represent more species than a randomly placed network, most recent expansions did increase representation more often, and for more groups, than would be expected as a result of just increases in the total area protected (Fig. 4). The analysis revealed that declared PAs performed better than randomly placed areas representing birds and plants since the 1976–1985 expansion (except for 1996–2005 in the case of plants), and also for mammals since 1986–1995. Contrastingly, the representation of herpetofauna only performed better than random placement of areas in the most recent decades (1986–1995 and 2006–2015 for reptiles, and 2006–2015 for amphibians). Threatened species have been captured better than by random networks for the last four decades. Notably, expansion during the last decade was the only period achieving better representation than expected by chance for all evaluated groups.

### Projected representation in climate change scenarios

When assessing the ability of PAs to adequately represent species in 2070 under a medium (RCP4.5) and a high emissions, business-as-usual (RCP8.5), climate-change scenarios, as estimated using projections of the species distribution models^34^, we observed that representation is expected to decrease for all taxa (Fig. 3), appreciably more in the high emissions scenario. Reptiles, the exception, were projected to result equally affected by the medium and high emissions scenarios. Mammals are expected to maintain the largest proportion of species represented in 2070 (25.4% in RCP4.5, 20.3% in RCP8.5) followed by birds (17.8% and 15.9%), while amphibians are expected to remain the least represented (7.9% and 5.7%), preceded by reptiles (~ 8.5%), and plants (10.9% and 9.8%). Threatened species representation is expected to drop to 6.2% in RCP4.5 and 4.6% in RCP8.5. Therefore, representation in scenario RCP8.5 is projected to be smaller for all groups, except reptiles, with differences among RCPs being more acute for threatened species (representation decreases a 19% more compared to RCP4.5), amphibians (16%), and mammals (15%).

The randomization analysis revealed that, in general, PAs created in last evaluated decade (2006–2015) were able to enhance representation for projected species’ bioclimatic niches for 2070 better than random networks in both climate-change scenarios (Fig. 4). Future projected representation for amphibians under RCP4.5 and reptiles under RCP8.5 scenarios were the exception. Contrastingly, threatened species projected distributions were not represented better than randomly expected in either RCP.

## Discussion

Protected Areas in the Tropical Andes have notably increased their coverage over the last 80 years, leading to a progressive improvement in species representation. Disentangling trends in the performance of PA expansions in species representation, our randomization analysis revealed that only PAs added in the last 30–40 years were, overall, better at representing species diversity than expected just as a consequence of the increasing area. Moreover, the high performance of the PA expansion over the last decade (2006–2015) was also found when considering future scenarios of climate change, for most species’ groups. These findings suggest that decisions related to locating PAs have become more strategic and efficient over time in boosting the adequacy of the representation of the diversity of species. However, the PA network still exhibits an unbalanced representation across species groups, marked by a lower representation of amphibians and reptiles, and a decrease in representation under climate change scenarios. Threatened species, a group of species that is arguably in need of receiving the highest protection, were found to include the lowest proportion of species reaching targets, including in climate change scenarios. Altogether, this indicates only partial success at representing species in the expansions of PAs in tropical Andean countries, and emphasizes the need for more strategic allocation of PAs in the future, taking into account the representation of multiple taxa, beyond the groups that have historically dominated conservation planning.

A temporal analysis of the performance of tropical Andean PAs in representing species over time allowed us to detect improvements in area-based conservation decision-making. It is possible that a departure from the old conservation paradigm to establish PAs on iconic yet often remote and unproductive lands^1,35^, in favor of focusing on areas with higher ecological value, has contributed to this result. Historically, PAs in the region were often sited to preserve high elevation watersheds and habitats such as *páramos* and iconic landscapes such as volcanos and recreation areas^36,37^. Over time, the aforementioned increase in species representation efficiency coincides with the declaration of PAs shifting towards lower elevations (Fig. 2) and areas with higher species richness such as lowland Amazonian forests and the Andean foothills (Figs. A.6 to A.16 in Appendix A). Regional^38^ and national documents assessing conservation needs and policy plans endorse the idea of increasing ecological representativeness in PA networks since this period. For instance, conservation directive plans from Peru and Ecuador place emphasis on representing habitats and biodiversity since the 1970s^38–43^, and similar concepts are found in plans from Colombia, Venezuela, and Bolivia in the late 1980s^44–46^. Another example comes from the Amazonian region, where most PAs were declared after the 70 s and often followed strategic planning, targeting biological representation at country and regional levels^39^. Finally, we note that even if the increase in performance compared to random scenarios is indicative of rising efficiency, this does not imply that decisions were optimal in terms of ecological representation efficiency. Expanding PA networks considering systematic conservation planning^47^ approaches has the potential to maximize network representativeness.

Additional research is needed to determine the extent to which strategic planning is behind the trend in increasing representation efficiency, or the relative role of alternative factors such as an increased demand from civil society to protect species-rich areas, or other national sociopolitical contexts such as funding availability and political will. We note that not all evaluated PAs are explicitly targeted at boosting species representation, as some are defensibly aimed at alternative objectives as varied as sparing land for ecosystem services or preserving sites of biocultural value. Investigating PA declaration and management planning documents in greater detail for declared intentionality at closing representation gaps may provide crucial insights to addressing this question but represents a daunting task because many of these documents are difficult to access. Equally interesting is testing if the burgeoning systematic conservation planning studies for the region^48–51^ are influencing more recent decision-making in PA declaration and contributing to improving PA performance over time.

Progressive PA expansions were often more efficient and represented better bird, mammal, and plant species than reptiles and amphibians, resulting in a PA network in which species representation is unbalanced across species groups. Herpetofauna was the worst represented group in 2015, consistent with regional^25^ and global assessments^16,52^. Most threatened species also remain under-represented, despite benefiting from efficient decisions of more recent PA additions. Overall, this unbalanced representation can be attributed to differences among species groups in terms of distribution patterns, geographic range sizes, and knowledge of their distributions. In the Tropical Andes, mammals and birds are better studied groups^53^ and have their distribution centers in the Amazonian lowlands, where their typically larger ranges tend to overlap^26^. This context allows a smaller number of well-chosen large PAs in the lowlands to simultaneously capture and represent many species. In contrast, amphibians and reptiles, and many threatened species, are mostly small-ranged species with little overlap among their distributions^26,54^. The distribution centers of these groups are characteristically displaced towards the Andean foothills and valleys^26,55^. This requires many small, scattered PAs, matching the specific localities in the Andes of these small-ranged and threatened species, to meet their representation needs efficiently^56^. Furthermore, progress towards an efficient protection of these species has been hindered by the historical lack of information on their spatial distribution^53,57^. Moreover, establishing multiple small state PAs in a matrix of agricultural lands with large human populations, characteristic of Andean landscapes, would incur very high management costs and social conflicts^55^. Under these circumstances, conservation in communal and private lands, although not analyzed here, plays an essential role in helping close representation gaps of small-ranged and threatened species^54,58^. Lastly, it is worth noting that herpetofauna and plants tended to have higher representation targets because they typically include small-range species. The targets of these species might have been more difficult to attain, contributing to their lower overall group representation.

The last PA expansion (2006–2015) performed better than random allocations in representing most species groups in future climate scenarios. The overall high efficiency of PAs declared in this decade can mainly be explained by their ability to capture areas that are projected to retain their species diversity in the future. Nevertheless, the strategic nature of this climate-efficient expansion is uncertain and should be explored further, as we were unable to detect national or regional plans for PAs prior to 2015 that explicitly consider improving ecological representation in present and future climates. In addition, the last PA expansion did not efficiently target projected ranges of threatened species under climate change scenarios. Incorporating systematic planning approaches is, therefore, crucial to inform forthcoming PA designations that can maximize present and future representation of threatened species and other taxa^59,60^. Notwithstanding, even with efficient PA designations, the overall representativeness is projected to decrease in future climate scenarios for all groups according to our projections. This result is consistent with studies in the Andes^61,62^, where species shift their distribution upslope in the search for colder climates, resulting in a shrinking of species’ ranges with elevation and, occasionally, even the complete disappearance of their bioclimatic niches. This drop in representation is projected to be more pronounced for reptiles and plants, which include many species that have limited dispersal capabilities to follow up with climate^34^. Therefore, although PAs are a valid adaptation strategy for species conservation under climate change, they are not a panacea and global mitigation policies remain an imperative task to avoid losses in species diversity^63^.

Our study has methodological caveats to mention. First, our randomization analysis preserved the shape and size of PAs to isolate the impact of the decisions made in terms of the sites protected. However, this approach might introduce biases in the random scenarios since some areas, particularly those close to the borders and the coast, could be prevented from being protected simply because the shape of PAs does not fit naturally. Future randomization algorithms may explore making PA shapes adaptable to other reserves and political or natural borders, or consider clustering aspects. Second, we did not consider historical downsizing and degazettement events in tropical Andean PAs^64^. Future research might consider historic PA shapes dynamically (i.e., accounting for shapes before downsize events) to improve estimates of species representation and efficiency over time. Third, accurately mapping past, current, and future species distributions has its challenges, particularly in the case of data-poor regions. We point out that, for each species, only one distribution model was built with all available occurrences and climate variables representative of the period 1960–1990, and these were evaluated against varying extents of PAs as the network expanded between 1937 and 2015. We did not build inter-decadal models due to a lack of finer temporal resolution climate data and comparable species occurrences. Likely, climate and species distributions slowly underwent changes between decades, but these were smaller and slower than those expected for the twenty-first century^65^. Finally, the large geographic scale and number of species included in the analysis hindered our ability to build SDMs with occurrences from species’ complete distributions. This limitation may result in an underestimation of species’ projected bioclimatic ranges^66^ and representation in PAs. To address this, our occurrence sampling method was designed to include data from areas beyond the countries assessed, which improved the representation of species' climatic variability. As a result, only few species with very large distributions could have been affected by this issue. Endemic and small ranged species, which are the most abundant in tropical Andean countries and are of higher conservation concern, are not expected to be affected.

## Conclusions

Our analysis for the Tropical Andes adds new evidence of the progress and impact of PA expansions, beyond simplistic metrics based on total area protected. For this region, we detected that more recent increases in PA coverage were accompanied with efficient increases in species coverage. Moreover, we identified further needs for the region, as most threatened species, amphibians, and reptiles remain highly underrepresented within PAs. In this context, the implementation of quantifiable and achievable regional targets for balancing representation across taxa will help design higher-impact PA expansions to halt species diversity loss^12,67^. Systematic conservation planning will be crucial in maximizing the efficiency of future PAs enhancing network representativeness and minimizing the impact of climate change on ecological representation^59,60^. Recently, the new Kunming-Montreal Global Biodiversity Framework set new international biodiversity goals and targets (at the moment, in the state of draft decision^68^), and specifies in its Target 3 that conserved area networks built by 2030 must be ecologically representative. We emphasize that it is crucial that proposed headline indicators to monitor progress in Target 3 include a target to track the ecological representativeness of networks^69^. Our analysis shows that indicators such as the change in representation of different groups of taxa and the performance of new protected areas in closing representation gaps are available to monitor this essential property of functioning conservation networks.

Lastly, we note that, besides enhancing species representation, there are other relevant criteria that guide the decision on what sites should be protected that do not necessarily provide the largest representation gains, such as securing the equitable access to natural assets^70^ or protecting intact forest landscapes^71^. Thus, the performance of PA location might vary with the criteria assessed, and additional evaluations might focus on these complementary aspects to broaden our understanding of trends in PA outcomes. Importantly, backward facing analyses such as ours provide useful insights on historical flaws and persisting needs, but also on success stories that may spur conservation efforts at this critical moment. Overall, the improved species representation in PAs over time herein described, should be understood as an incentive in the international and national conservation agendas for ongoing efforts to continue improving the performance of area-based conservation in a rapidly changing world.

## Methods

### Protected-area networks in tropical Andean countries

This study considers national terrestrial PAs in the five tropical Andean countries (Fig. 1, Appendix B), excluding islands. The regional PA network of the five countries comprised 274 reserves in 2015, occupying 14.7% of the terrestrial area (Fig. 2a). Initiated in 1937, the network has grown rapidly and non-uniformly since, with a peak in expansion between 1985 and 2000 and a deceleration in the last decade (Fig. 2b). By 1985, half the total area protected today had already been incorporated into the network. There has been a downward shift towards protecting areas at lower elevations over time (Fig. 2c).

As the aim was to assess trends in the performance of representing species of a globally relevant conservation network, we conducted analyses on the regional network, as integrated by areas from any of the five countries. The list of PAs evaluated included only those that are part of countries’ state-managed networks. In the region, conservation figures with governance other than national (private, regional, municipal PAs) exist, but they presently cover a small percentage of the countries. However, these areas are growing in the region^72^ and may play an important role in closing representation gaps^58,73^. We did not include these areas in our assessment because spatial data availability for these is highly heterogeneous across the region. The conservation networks analyzed may include PAs classified within any of the IUCN management categories (I–VI).

### Species distribution data

Representation was evaluated for 16,510 species, made up of 1810 birds, 197 mammals, 699 amphibians, 452 reptiles, and 13,352 plants (Appendix C). Of these, 56 are Critically Endangered, 246 are Endangered, and 501 are Vulnerable^32^.

Species occurrences were compiled from online data facilities, museums, and literature for amphibians, reptiles, mammals, birds, and plants (including mosses, ferns and allies, gymnosperms, and flowering plants) in the Tropical Andes. To ensure an adequate representation of species’ bioclimatic niches, occurrence data was obtained for an area that comprised the five countries under study and an expanded area including portions of neighbor countries. Occurrence data was checked through automated evaluation and expert criteria to remove geographic and taxonomic errors (see Appendix A for a full description of data sampling and correction methods).

### Species distribution models

Species distribution models (SDM)^74^ were used to represent species’ bioclimatic niches and to forecast geographic shifts under climate change scenarios. SDMs were generated in R using the BIOMOD2 package^28^ as ensembles of three modelling techniques: Maxent^31^, random forests^29^, and boosted regression trees^30^. Models were built using five Worldclim1.4 bioclimatic variables as predictors^75^: mean diurnal range, temperature seasonality, maximum temperature of the warmest month, annual precipitation and precipitation of the driest month. These are variables with potential biological relevance to describe species’ bioclimatic niche, and were kept after removal of correlated bioclimatic variables. SDMs were produced at a ~ 5 km^2^ resolution for species with at least 25 unique occurrences^76^. To maximize the inclusion of small-ranged species (herein, species with an extent of occurrence ≤ 500 km^2^) with few occurrences (9–25), we built ensembles of small models (ESM)^77^ using the same three modelling techniques (n = 5394 species). ESMs are ensembles of bivariate models built with all pairwise combinations of the predictors and are more robust than regular SDMs when modelling small-ranged species with few occurrences^77^. To minimize the transference of the spatial bias in collections to models, we used a background where the location of 10,000 points simulated the bias in occurrences^78^. Ensemble bioclimatic models were obtained by a weighted combination of single-technique models based on their performance according to the true-skill statistic (TSS) evaluation metric. Only single-technique models with TSS > 0.8 and area under the receiver operating characteristic curve (AUC) > 0.8 were used to build the ensembles. Underperforming ensemble models (TSS < 0.7 or AUC < 0.8) were discarded. In total, 2007 species were discarded due to underperforming evaluation. Ensemble models had a mean TSS value of 0.942 (95% range: 0.773–1.000), and a mean AUC value of 0.987 (0.837–1.000). We followed Thornhill et al.^79^ to restrict the final maps to areas that are both climatically suitable and geographically close to each species known occurrences. Model outputs were transformed to presence/absence values using the “10 percentile presence” threshold^80^. This is a conservative threshold that excludes occurrences with the smallest suitability, seeking to reduce commission errors^81^. Additional modelling details in Appendix A.

### Species representation assessment

We assessed species representation by calculating the area of each species distribution (i.e., their SDM projected under current or 2070 climatic conditions) covered by PAs, and contrasting this coverage with species-specific representation targets^27^. Species representation targets are often used in gap analyses^1,27,82^, and where designed as a measure of adequacy to reflect species protection needs based on ecological and conservation criteria^14^. Building on Rodrigues et al.^27^ and Butchart et al.^52^, targets were log-scaled between 100% of the distribution for species with a modelled range of 1000 km^2^ and smaller, and 20% for those with ranges larger than 55,000 km^2^ (upper quartile of the species, but see Appendix A for target sensitivity tests). Small-ranged species received a higher target due to their higher vulnerability to extinction from adverse natural or anthropogenic events^83^. Species’ representation target values used in the assessment are included in Appendix C.

We evaluated the representation of groups (taxonomic and threat status) by calculating the percentage of species meeting their targets in the PA network every decade over its expansion. We chose to evaluate representativeness using species representation targets because these provide a metric to assess the adequacy of representation that is critical towards the goal of species’ persistence. Using representation targets in gap analyses improves the accountability and defensibility of the process when compared with simpler metrics based on direct coverage (i.e., average coverage of species distributions)^1^.

### Randomization analysis of species representation over time

To evaluate performance in species representation over time of expanding PA networks, we conducted a randomization analysis to test whether PAs declared in each decade resulted in larger gains in representation than would be expected if the same PAs had been placed at random locations^33^. This analysis assesses whether the increase in representation is just a consequence of the increase in the total area protected or whether the chosen PA configurations (encompassing aspects such as the number of PAs and their location, shape, and size) significantly enhanced that contribution more than expected by chance. We produced 100 randomized PA networks for each decade of expansion (1937–1945, 1946–1955, 1956–1965, 1966–1975, 1976–1985, 1986–1995, 1996–2005, and 2006–2015), where the location of all PAs declared within the period in the five countries were randomly moved by translation, flipping, and rotation throughout the study area whilst preserving their spatial attributes other than location. Randomly allocated PAs were not allowed to overlap with each other, with the real location of existing PAs declared during previous decades, or the study area border so that the total area under protection was kept constant. Previous analyses have shown that the random allocation of smaller and numerous PAs leads to a higher ecological representation than the same area organized in fewer and larger units^19^, demonstrating that maintaining network characteristics such as PA number and size are key to assess performance. Therefore, our randomization approach preserved the number, size, and shape of declared PAs (see Appendix A for a full description of the randomization framework and a discussion of the implications of this decision). We compared species representation in each randomly simulated network with representation within the actual PAs declared that decade. The observed representation was estimated greater than expected by chance (P ≥ 0.05) if the observed value fell within the 5% upper tail of the distribution of random scores.

### Representation in climate change scenarios

We calculated the representation of groups within the complete 2015 PA network considering projected future potential distributions for the year 2070 in two climate-change scenarios (or Representative Concentration Pathways [RCP]): RCP4.5, a scenario of intermediate mitigation of greenhouse gas emissions, and RCP8.5, a business-as-usual high emissions scenario^65^.

For each scenario, we considered two general circulation models (GCMs), HadGEM2-ES and MPI-ESM-LR, which capture rainfall over the Amazon basin better than others, especially over the dry season^84^. One final projection was obtained for each RCP by averaging estimates from the two GCMs. Projections of species bioclimatic niche were transformed to presence/absence values using the “10 percentile presence” threshold^80^, consistent with the values used to threshold current-condition maps. Future projections were modified to apply dispersal restrictions using thresholds that varied across taxonomic groups. Specifically, we assigned a value of zero to areas falling outside of the distance threshold, using the edge of distance-refined binary models built with present variables as a baseline (see section on dispersal restrictions in Appendix A for more detail). Then, we contrasted projected distributions with PAs to estimate representation based on the same targets used to assess current coverage. We also extended the randomization analysis to future scenarios to estimate whether the last network expansion (2006–2015) represents species projected distributions better than expected by chance. We restricted this analysis to PAs from the last decade because these areas were created during the period in which awareness for future representation became increasingly relevant to conservationists and planners.

## Supplementary Information

Supplementary Information 1.

Supplementary Information 2.

Supplementary Information 3.

Supplementary Information 4.

## Data Availability

All data and scripts used in this paper are available upon reasonable request to the corresponding author.

## References

[1] Possingham HP, Wilson KA, Andelman SJ, Vynne CH, Groom MJ, Meffe GK, Carroll CR (2006). Protected areas. Goals, limitations, and design. Principles of Conservation Biology.

[2] Marquet PA, Lessmann J, Shaw MR, Lovejoy TE, Hannah L (2019). Protected-area management and climate change. Biodiversity and Climate Change: Transforming the Biosphere.

[3] Geldmann J, Manica A, Burgess ND, Coad L, Balmford A (2019). A global-level assessment of the effectiveness of protected areas at resisting anthropogenic pressures. PNAS.

[4] Potapov P (2017). The last frontiers of wilderness: Tracking loss of intact forest landscapes from 2000 to 2013. Sci. Adv..

[5] Cazalis V (2020). Effectiveness of protected areas in conserving tropical forest birds. Nat. Commun..

[6] Dudley N, Mansourian S, Stolton S, Suksuwan S (2010). Do protected areas contribute to poverty reduction?. Biodiversity.

[7] Dudley N, Stolton S (2010). Arguments for Protected Areas.

[8] CBD. *Strategic Plan for Biodiversity 2011–2020, Including Aichi Biodiversity Targets*. http://www.cbd.int/sp/ and http://www.cbd.int/decision/cop/?id=12268 (2010).

[9] UNEP-WCMC & IUCN. *Protected Planet: The World Database on Protected Areas (WDPA)*. www.protectedplanet.net. Accessed October 2022 (2022).

[10] Watson JEM (2016). Persistent disparities between recent rates of habitat conversion and protection and implications for future global conservation targets. Conserv. Lett..

[11] Díaz, S. *et al*. *Summary for Policymakers of the IPBES Global Assessment Report on Biodiversity and Ecosystem Services*. (2019).

[12] Barnes MD, Glew L, Wyborn C, Craigie ID (2018). Prevent perverse outcomes from global protected area policy. Nat. Ecol. Evol..

[13] Visconti P (2019). Protected area targets post-2020. Science.

[14] Kukkala AS, Moilanen A (2013). Core concepts of spatial prioritisation in systematic conservation planning. Biol. Rev..

[15] Joppa LN, Pfaff A (2009). High and Far: Biases in the location of protected areas. PLoS ONE.

[16] Maxwell SL (2020). Area-based conservation in the twenty-first century. Nature.

[17] CBD. *CoP 7 Decision VII/30. Strategic Plan: Future Evaluation of progress*. 12 https://www.cbd.int/doc/decisions/cop-07/cop-07-dec-30-en.pdf (2004).

[18] Venter O (2017). Bias in protected-area location and its effects on long-term aspirations of biodiversity conventions. Conserv. Biol..

[19] Kuempel CD, Chauvenet ALM, Possingham HP (2016). Equitable representation of ecoregions is slowly improving despite strategic planning shortfalls. Conserv. Lett..

[20] Barr LM, Watson JEM, Possingham HP, Iwamura T, Fuller RA (2016). Progress in improving the protection of species and habitats in Australia. Biol. Conserv..

[21] Hoffmann S, Irl SDH, Beierkuhnlein C (2019). Predicted climate shifts within terrestrial protected areas worldwide. Nat. Commun..

[22] Hannah L (2008). Protected areas and climate change. Ann. N. Y. Acad. Sci..

[23] Thomas CD, Gillingham PK (2015). The performance of protected areas for biodiversity under climate change. Biol. J. Lin. Soc..

[24] Ramirez-Villegas J (2014). Using species distributions models for designing conservation strategies of Tropical Andean biodiversity under climate change. J. Nat. Conserv..

[25] Bax V, Francesconi W (2019). Conservation gaps and priorities in the Tropical Andes biodiversity hotspot: Implications for the expansion of protected areas. J. Environ. Manage..

[26] Jenkins CN, Pimm SL, Joppa LN (2013). Global patterns of terrestrial vertebrate diversity and conservation. PNAS.

[27] Rodrigues ASL (2004). Global gap analysis: Priority regions for expanding the global protected-area network. Bioscience.

[28] Thuiller, W., Georges, D., Engler, R. & Breiner, F. *biomod2: Ensemble Platform for Species Distribution Modeling*. (2015).

[29] Breiman L (2001). Random forests. Mach. Learn..

[30] Friedman JH (2001). Greedy function approximation: A gradient boosting machine. Ann. Stat..

[31] Phillips SJ, Anderson RP, Dudík M, Schapire RE, Blair ME (2017). Opening the black box: An open-source release of Maxent. Ecography.

[32] IUCN. *The IUCN Red List of Threatened Species*. (2017).

[33] Gotelli, N. J. & Graves, G. R. *Null Models in Ecology*. (1996).

[34] Araújo MB, Pearson RG (2005). Equilibrium of species’ distributions with climate. Ecography.

[35] Watson JEM, Grantham HS, Wilson KA, Possingham HP, Ladle RJ, Whittaker RJ (2011). Systematic conservation planning: Past, present and future. Conservation Biogeography.

[36] Bevilacqua M (2003). Áreas protegidas y conservación de la diversidad biológica. Biodivers. Venezuela.

[37] Franco P, Saavedra-Rodríguez CA, Kattan GH (2007). Bird species diversity captured by protected areas in the Andes of Colombia: A gap analysis. Oryx.

[38] Barzetti, V. *Parks and Progress: Protected Areas and Economic Development in Latin America and the Caribbean*. (1993).

[39] Schulman L (2007). Amazonian biodiversity and protected areas: Do they meet?. Biodivers. Conserv..

[40] Dourojeanni MJ (2018). Áreas naturales protegidas e investigación científica en el Perú. Rev. For. Perú.

[41] Rodriguez L, Young K (2000). Biological diversity of Peru: Determining priority areas for conservation. Ambio.

[42] Ministerio del Ambiente & SERNANP. *Plan Director de las Áreas Naturales Protegidas (Estrategia Nacional)* (2009).

[43] Cuesta-Camacho, F. *et al*. *Identificación de Vacíos y Prioridades de Conservación Para la Biodiversidad Terrestre en el Ecuador Continental*. http://protectedareas.info/upload/document/ecuador_terrestrial_gap_analysis.pdf (2006).

[44] Naveda S (1997). Evaluación del grado de representatividad ecológica y geográfica del Sistema de PN de Venezuela al norte del Orinoco: Anteproyecto. Rev. Geog. Venez..

[45] Araujo, N., Müller, R., Nowicki, C. & Ibisch, P. L. *Prioridades de conservación de la biodiversidad de Bolivia *(editorial FAN, 2010)

[46] Arango, N. *et al*. *Vacíos de Conservación del Sistema de Parques Nacionales Naturales de Colombia desde una Perspectiva Ecorregional*. https://wwflac.awsassets.panda.org/downloads/vacios_de_conservacion.pdf (2003).

[47] Margules CR, Pressey RL (2000). Systematic conservation planning. Nature.

[48] Sarkar S, Sánchez-Cordero V, Londoño MC, Fuller T (2009). Systematic conservation assessment for the Mesoamerica, Chocó, and Tropical Andes biodiversity hotspots: A preliminary analysis. Biodivers. Conserv..

[49] Lessmann J, Muñoz J, Bonaccorso E (2014). Maximizing species conservation in continental Ecuador: A case of systematic conservation planning for biodiverse regions. Ecol. Evol..

[50] Young BE (2009). Using spatial models to predict areas of endemism and gaps in the protection of Andean slope birds. Auk.

[51] Fajardo J, Lessmann J, Bonaccorso E, Devenish C, Muñoz J (2014). Combined use of systematic conservation planning, species distribution modelling, and connectivity analysis reveals severe conservation gaps in a megadiverse country (Peru). PLoS ONE.

[52] Butchart SHM (2015). Shortfalls and solutions for meeting national and global conservation area targets. Conserv. Lett..

[53] Pimm SL (2014). The biodiversity of species and their rates of extinction, distribution, and protection. Science.

[54] Swenson JJ (2012). Plant and animal endemism in the eastern Andean slope: Challenges to conservation. BMC Ecol..

[55] Lessmann J, Fajardo J, Bonaccorso E, Bruner A (2019). Cost-effective protection of biodiversity in the western Amazon. Biol. Conserv..

[56] Rodrigues ASL, Gaston KJ (2001). How large do reserve networks need to be?. Ecol. Lett..

[57] Reyes-Puig C (2017). Diversity, threat, and conservation of reptiles from continental Ecuador. Amphib. Reptile Conserv..

[58] Shanee S (2017). Protected area coverage of threatened vertebrates and ecoregions in Peru: Comparison of communal, private and state reserves. J. Environ. Manage..

[59] Kujala H, Moilanen A, Araújo MB, Cabeza M (2013). Conservation planning with uncertain climate change projections. PLoS ONE.

[60] Hannah L (2020). 30% land conservation and climate action reduces tropical extinction risk by more than 50%. Ecography.

[61] Velásquez-Tibatá J, Salaman P, Graham CH (2013). Effects of climate change on species distribution, community structure, and conservation of birds in protected areas in Colombia. Reg. Environ. Change.

[62] del Avalos VR, Hernández J (2015). Projected distribution shifts and protected area coverage of range-restricted Andean birds under climate change. Glob. Ecol. Conserv..

[63] Warren R (2013). Quantifying the benefit of early climate change mitigation in avoiding biodiversity loss. Nat. Clim. Change.

[64] Golden Kroner R (2021). COVID-era policies and economic recovery plans: Are governments building back better for protected and conserved areas?. PARKS.

[65] Stocker TF, IPCC (2013). Summary for Policymakers. Climate Change 2013: The Physical Science Basis. Contribution of Working Group I to the Fifth Assessment Report of the Intergovernmental Panel on Climate Change.

[66] Chevalier M, Zarzo-Arias A, Guélat J, Mateo RG, Guisan A (2022). Accounting for niche truncation to improve spatial and temporal predictions of species distributions. Front. Ecol. Evol..

[67] Watson JEM (2016). Bolder science needed now for protected areas. Conserv. Biol..

[68] CBD. *Kunming-Montreal Global Biodiversity Framework, Draft Decision Submitted by the PRESIDENT*. (2022). CBD/COP/15/L.25. https://www.cbd.int/doc/c/e6d3/cd1d/daf663719a03902a9b116c34/cop-15-l-25-en.pdf

[69] CBD (2022). Report of the Expert Workshop on the Monitoring Framework for the Post-2020 Global Biodiversity Framework.

[70] Chaplin-Kramer R (2022). Mapping the planet’s critical natural assets. Nat. Ecol. Evol..

[71] Watson JEM (2018). The exceptional value of intact forest ecosystems. Nat. Ecol. Evol..

[72] Elbers, J. *Las Áreas Protegidas de América Latina: Situación Actual y Perspectivas PARA el Futuro* (2011).

[73] Miller DC, Nakamura KS (2018). Protected areas and the sustainable governance of forest resources. Curr. Opin. Environ. Sustain..

[74] Guisan A, Zimmermann NE (2000). Predictive habitat distribution models in ecology. Ecol. Model..

[75] Hijmans RJ, Cameron SE, Parra JL, Jones PG, Jarvis A (2005). Very high resolution interpolated climate surfaces for global land areas. Int. J. Climatol..

[76] van Proosdij ASJ, Sosef MSM, Wieringa JJ, Raes N (2016). Minimum required number of specimen records to develop accurate species distribution models. Ecography.

[77] Breiner FT, Guisan A, Bergamini A, Nobis MP (2015). Overcoming limitations of modelling rare species by using ensembles of small models. Methods Ecol. Evol..

[78] Phillips SJ (2009). Sample selection bias and presence-only distribution models: Implications for background and pseudo-absence data. Ecol. Appl..

[79] Thornhill AH (2017). Spatial phylogenetics of the native California flora. BMC Biol.

[80] Radosavljevic A, Anderson RP (2014). Making better Maxent models of species distributions: Complexity, overfitting and evaluation. J. Biogeogr..

[81] Kershaw F (2013). Informing conservation units: Barriers to dispersal for the yellow anaconda. Divers. Distrib..

[82] Venter O (2014). Targeting global protected area expansion for imperiled biodiversity. PLoS Biol..

[83] Gaston KJ (2003). The Structure and Dynamics of Geographic Ranges.

[84] Yin L, Fu R, Shevliakova E, Dickinson RE (2013). How well can CMIP5 simulate precipitation and its controlling processes over tropical South America?. Clim. Dyn..

