# Supplementary material for: The performance of protected-area expansions in representing tropical Andean species: past trends and climate change prospects
Source: Sci Rep. 2023 Jan 18;13:966. doi: 10.1038/s41598-022-27365-7 (PMC9849396; doi:10.1038/s41598-022-27365-7)
Supplement: Supplementary file 1 — Supplementary Information 1. [file 41598_2022_27365_MOESM1_ESM.pdf]

Appendix A - Extended methods and results

The performance of protected-area expansions in representing tropical Andean species: trends and climate change prospects

Javier Fajardo, Janeth Lessmann, Christian Devenish, Elisa Bonaccorso, Ángel M. Felicísimo, Fernando J. M. Rojas-Runjaic, Haidy Rojas, Miguel Lentino, Jesús Muñoz, Rubén G. Mateo

Table of Contents

1. Protected area data ... 2
2. Species data ... 3
Lists of species ... 3
Species occurrence data ... 3
Biases and known problems of occurrence data ... 5
3. Species distributions models ... 7
Modeling techniques ... 7
Refining species distribution models ... 10
Future scenarios ... 11
Dispersal restrictions in future scenarios ... 11
Known limitations of species distribution models ... 12
4. Set of analyzed species ... 13
5. Randomization analysis – Expanded details ... 14
Framework and algorithm description ... 14
Framework results ... 16
Sensitivity analysis ... 18
6. Patterns of not-represented species with decadal PA expansions ... 20
All species (Fig. A.6) ... 21
Threatened species (Fig. A.7) ... 22
Birds (Fig. A.8) ... 23
Mammals (Fig. A.9) ... 24
Amphibians (Fig. A.10) ... 25
Reptiles (Fig. A.11) ... 26
Plants (Fig. A.12) ... 27
Gymnosperms (Fig. A.13) ... 28
Flowering plants (Fig. A.14) ... 29
Bryophytes (Fig. A.15) ... 30
Ferns and allies (Fig. A.16) ... 31
7. References ... 32

1. Protected area data

Included protected areas (PAs) are part of countries’ state-managed systems, which are: *Sistema Nacional de Áreas Protegidas* (SNAP, Bolivia), *Sistema Nacional de Áreas Naturales Protegidas por el Estado* (SINANPE, Peru), *Patrimonio de Áreas Naturales del Estado* (PANE, Ecuador), *Sistema Nacional de Áreas Protegidas* (SINAP, Colombia) and *Instituto Nacional de Parques* (INPARQUES, Venezuela). We downloaded data for each country in shapefile format. Find the complete list in Appendix B.

We have not included area categories that do not imply operative management conservation management. In the case of Peru, we did not include *Zonas Reservadas* (Reserved Zones) in the analyses because these areas, have not yet being declared as formal conservation areas. In the case of Venezuelan Tepuis National Monuments, we only considered their *Zonas de Protección Integral* (ZPI; Protection Integral Zones). These are defined as areas above 800 m.a.s.l., located within larger "polygonal" areas [1]. Polygonal areas have a characteristic square or rectangular shape that does not follow the natural limits of geographies. We only included ZPI because these are the areas where restrictive activities and the extraction of biological resources is not permitted [1].

We also note that the protected areas considered here for Venezuela result in an extent that differs substantially from some estimations, such as the reporting from ProtectedPlanet.net (56.8% of terrestrial coverage, accessed Nov 2020). Indeed, mismatches such as ours are considerable and common in the literature on the total area protected in Venezuela [2]. The reason behind these mismatches is that many estimations include areas within the *Áreas Bajo Régimen de Administración Especial* (ABRAE) system when calculating the area under protection. ABRAE is a system that includes all protected areas in INPARQUE among other areas. In fact, most ABRAE areas have been matched to IUCN categories of protected-area management and are included in the World Database of Protected Areas (ProtectedPlanet.net) dataset for Venezuela. Nonetheless, ABRAE includes many areas that do not include biodiversity conservation among its objectives. Instead, they range from regulating forestry and agricultural land to reserving areas suitable for the construction of dams [2].

2. Species data

2.1. Lists of species

Lists of species were obtained for the five countries from these sources: amphibians from Amphibian species of the world [3], birds from the South American Classification Committee (SACC)[4], mammals from the IUCN Red List database of species [5], reptiles from The Reptile-Database [6], and plants from the Botanical Information and Ecology Network (BIEN 3.4) [7, 8]. We followed the taxonomy in those references.

We did not analyze flying mammals or rodents from the Critecidae family because we were not able to compile a comprehensive, spatially balanced and taxonomically robust occurrence database for the five countries. Species threat status was obtained from the IUCN Red List of threatened species [9]. The plant species list was inspected for introduced and cultivated species with the ‘taxize’ and ‘originr’ R packages [10, 11]. Species tagged as cultivated, introduced or invasive in the study area in any of the databases were removed.

2.2. Species occurrence data

Species occurrences were downloaded from a wide range of sources. A large proportion was obtained from online open-access data-portals such as the Botanical Information and Ecology Network for plants (BIEN 3+ database; http://bien.nceas.ucsb.edu/bien/biendata/bien-3/), the Global Environmental Information Facility (GBIF; http://www.gbif.org/), Sistema de Información sobre Biodiversidad de Colombia (SIB Colombia; https://www.sibcolombia.net/), eBIRD (Cornell Lab of Ornithology; http://ebird.org), VertNet (http://www.vertnet.org), the Mammal Networked Information system (MANIS; http://manisnet.org/), IABIN (http://www.oas.org/en/sedi/dsd/iabin/), and SpeciesLink (http://splink.cria.org.br). A list of institutions that contributed through these data portals can be found at Appendix D.

Downloaded GBIF data DOIs:
doi.org/10.15468/dl.s0hqv2; doi.org/10.15468/dl.rpidwc; doi.org/10.15468/dl.slyjxf; doi.org/10.15468/dl.y8hlqe; doi.org/10.15468/dl.ihf1fd; doi.org/10.15468/dl.e7ym01; doi.org/10.15468/dl.i9vhxq; doi.org/10.15468/dl.d1j5hq.

In addition, we obtained occurrences from private databases and natural science museums. The databases included the Bolivian large and medium-size mammals database [12], Registro Nacional de Colecciones Biológicas of Venezuela (RECOB, Venezuela, www.diversidadbiologica.minamb.gob.ve/recob.php; Institutions sharing data in RECOB are also listed in Appendix D), and Biomap (Colombia, http://www.biomap.net). The following museums contributed species records from their database: Museo de Zoología de la Universidad Tecnológica Indoamérica (MZUTI, Quito, Ecuador), colección de anfibios y reptiles del Museo de Historia Natural La Salle (MHNLS, Caracas, Venezuela), Fundación William H. Phelps (www.fundacionwhphelps.org; Caracas, Venezuela), Field Museum's Rapid Biological Inventories (Chicago, USA), the William E. Duellman field series [13], Universidad de Antioquia (Medellín, Colombia), Centro Geoespacial para la Biodiversidad at Museo de Historia Natural Noel Kempff Mercado (http://www.museonoelkempff.org/cgb; Santa Cruz, Bolivia), and Museo Nacional de Ciencias Naturales (MNCN; Madrid, Spain). The following experts shared additional occurrences: Christian Devenish, Stefan Löthers, Jörn Köhler, José Manuel Padial, Dirk Embert, Jaime Culebras, Mariana Delgado, Juan Manuel Carvajalino Fernández, Jesús N. Pinto-Ledezma, Elkin Noguera, Héctor Ramírez, Jetzabel Gross, Oscar Quiroga, Ana M. Umaña, Juanita Saldana and Mauricio Álvarez. Additional records were taken from different scientific publications, books and NGO reports (citations in Appendix D).

Occurrences with coordinates were obtained for the bounding-box delimited by -82/-55 degrees of longitude and -25/13 degrees of latitude (therefore, including records in the neighbor countries Panama, Guyana and Brazil, a procedure intended at sampling better the environmental niche of the species and at not biasing models at the borders of the study area, following recommendations for species distribution modeling [14]). Data from online databases and museums were only used when coordinates were provided and there were no known coordinate issues reported. In the case of species records taken from bibliography, coordinates were sometimes obtained using the Geonames resolver (www.geonames.org) for localities described in detail, or extracted from the published map when coordinates were not provided.

All records were carefully checked following automated processes, and suspicious occurrences were removed. The automated processes to identify problematic occurrences

included, where possible, the spatial cross-validation of recorded first and second-level administrative information with administrative maps; occurrences with a mismatch greater than 0.15 degrees (~30 km at the equator) were removed. We also excluded records identified as invalid, with plain zero coordinates, or in a buffer of 0.15 degrees diameter around country and province centroids, following Maldonado et al. [15]. In addition, vertebrate data was manually checked for errors. As part of this manual process, occurrences were compared to IUCN ranges [16, 17], and suspicious records in disagreement with reported ranges were sometimes deleted following expert criteria.

The curated database included data for 68,617 species, from which 50,100 species were not included in the analyses because they had a number of records insufficient for modeling (see below).

2.3. Biases and known problems of occurrence data

Natural history collections and databases of species occurrences are prone to temporal and spatial biases and errors [18]. These issues are also present in the tropical Andes region, with some regions substantially less sampled than others, such as remote parts of the Amazon and Andean foothills (Figure A.1) [19]. In this context, further sampling efforts are critically needed in the tropical Andean countries to reduce the geographic and taxonomic bias of species records that could lead in the future to more refined results than those obtained here.

Regarding errors in geo-referencing, the automated verification and depuration processes, combined with manual verification of vertebrate data, do not replace fully manual verification by specialists. However, the methods followed were able to detect a large number of problematic records and led to substantial improvement of the quality of the database, and can be considered best practice taking into account the spatial scale of our analysis and the large number of species evaluated.

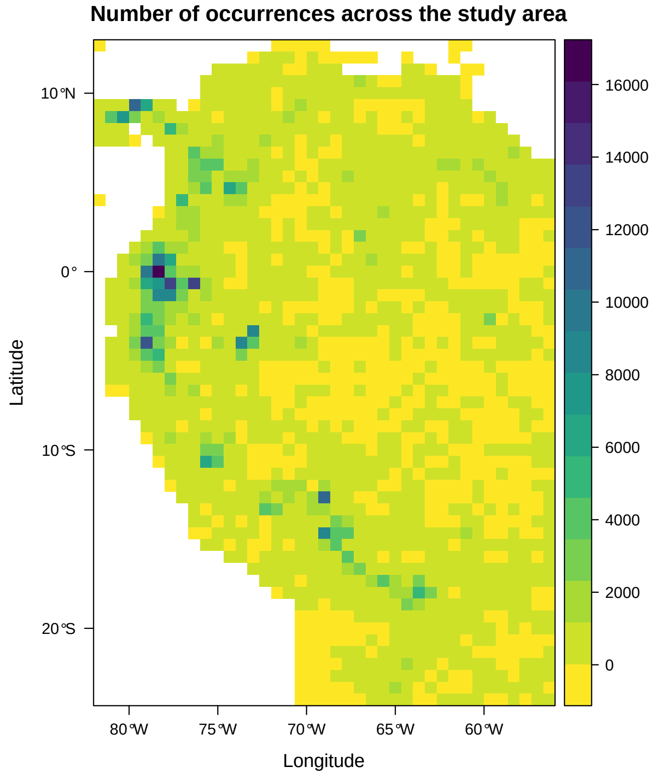

Figure A.1: Number of species occurrences used to build species distribution models. The numbers refer to unique occurrences kept after the validation process, counted in a grid of 0.6 decimal degree resolution. Notice that occurrences were not restricted to the five countries evaluated. Figure created in R v4.0.0 (https://cran.r-project.org/).

3. Species distribution models

3.1. Modeling techniques

Species occurrences were used to map species bioclimatic niches [20]. Species distribution models (SDMs) were produced using the package BIOMOD2 [21] in R [22] as ensembles of three different techniques: Maxent [23], Random Forest [24] and Boosted Regression Trees [25]. Models were built using bioclimatic variables from Worldclim 1.4 as ecological predictors [26]. We did not consider variables "mean temperature of wettest quarter" (bio 8), "mean temperature of driest quarter" (bio 9), "precipitation of warmest quarter" (bio 18), and "precipitation of coldest quarter" (bio 19), because they display unrealistic patterns over the study area (Fig. A.2) [27]. These variables display an abrupt variation of temperature and precipitation at some sections of the tropical Andes, which contrasts with the smooth variation that naturally describe these variables that is the result of combining, by definition, temperature and precipitation data. To avoid multicollinearity, we conducted a Pearson correlation analysis to remove variables with a coefficient greater than 0.8. From each pair of correlated variables, we kept the variable that was potentially more relevant to characterize species macroclimatic niches. This left a set of 5 variables that was used to build the models: "mean diurnal range" (bio 2), "temperature seasonality" (bio 4), "maximum temperature of the warmest month" (bio 5), "annual precipitation" (bio 12) and "precipitation of the driest month" (bio 14). Models were produced using environmental raster data with a resolution of 0.04167 degrees (~5 km at the equator).

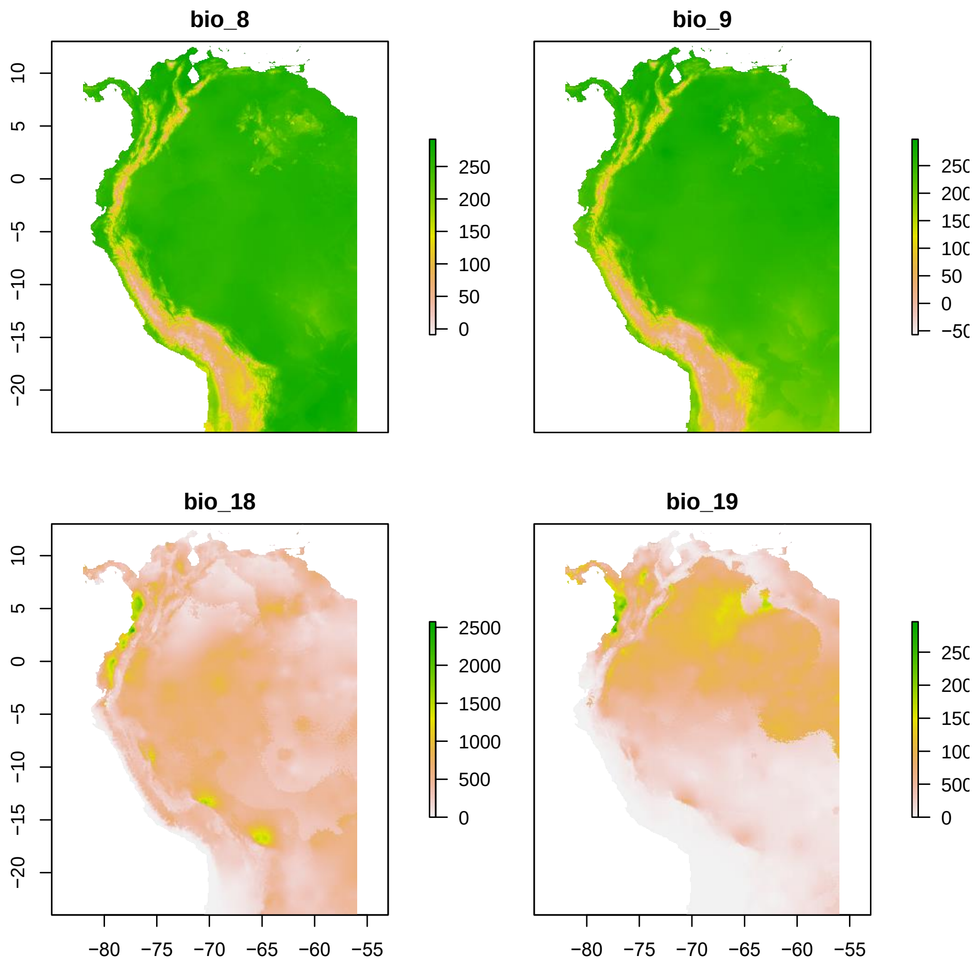

Figure A.2: Unrealistic patterns in bioclimatic variables over the tropical Andean countries. The pattern is found in the four variables that, by definition, combine temperature and precipitation data, because months or quarters with maximum or minimum values may vary between one pixel and its neighbors. It is characterized by drastic shifts in the variable value from some pixels over its neighbors, resulting in "waves" or "hard borders" that do not correspond with the smoother natural variation of these variables over landscapes. The pattern is more visible in bio 18 and bio 19, but notice the absence of a natural gradient in the green regions to the South in bio 8 and bio 9. Figure created in R v4.0.0 (https://cran.r-project.org/).

We used 10,000 points as background data in the modeling process [28]. To avoid overfitting, we used a bias grid (i.e., a raster layer containing a geographic bias comparable to that present in species occurrences) to sample background points. This has been shown to

offset the bias in occurrences, and thus render model outputs less affected by bias sampling [29]. In tropical regions, species occurrences are typically recorded more often in accessible areas near towns, roads or rivers [30]. Therefore, we used the human footprint raster layer [31] as a bias grid to sample background points [32]. The human footprint maps eight human pressures on the environment, including human population density, night-time lights, roads, railways, and navigable rivers [31]. To illustrate the relationship between the human footprint and sampling bias, Figure A.3 compares the number of occurrences points in a 25 km^2 grid and the human footprint layer, showing a visible correspondence.

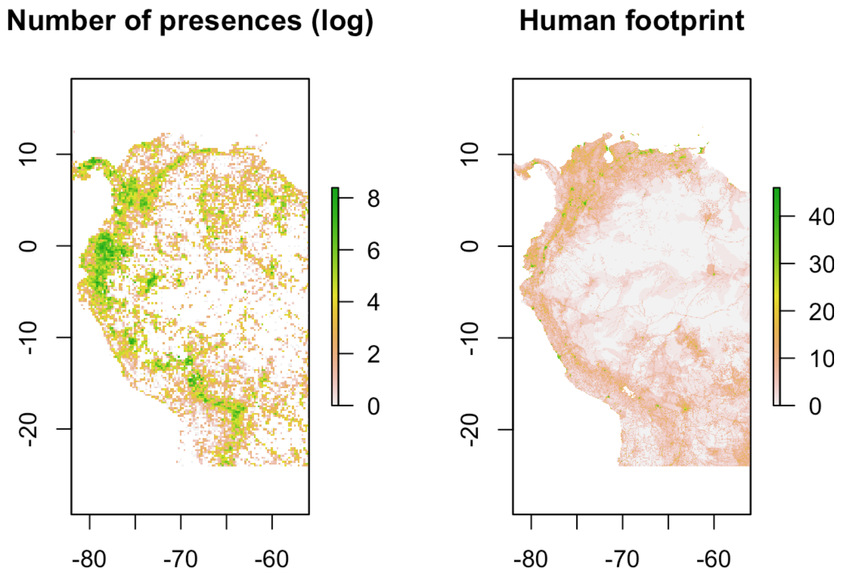

Figure A.3: Number of total occurrences (log) in the study area (left, 25km^2 grid) and human footprint index (right). Figure created in R v4.0.0 (https://cran.r-project.org/).

Most species' bioclimatic niches were modelled using regular ensemble SDMs (11,116 spp) with 25 or more occurrences [33]. However, with the objective of maximizing the inclusion of small-ranged, rare, and threatened species, typically with few species records, and often occurring at very close localities, some species (5394 spp, ~33 %) with restricted range (extent of occurrence $\leq$ 500 km^2, calculated from our species records) and few occurrences (9-25 records) were modelled using the alternative technique of Ensembles of Small Models (ESM) [34]. ESMs are ensembles of bivariate models built with all pair-

combination of the ecological predictors, and are more robust than regular SDMs when modelling small-ranged species with few occurrences [34]. Species with an insufficient number of occurrences to build SDM or ESM were not analyzed (n = 50,100). Exceptionally, ESMs were built with 0.008333 degree-resolution bioclimatic variables (~1 km at the equator) when the usual resolution did not allow maintaining the minimum number of unique records (577 spp, ~3 %). In these cases, the resulting maps were resampled at the 0.04167 degree-resolution. Adopting these alternative methods made possible the inclusion of species that are often in great need of conservation action.

Ensemble models were calculated using the proportional weighted means of probabilities option, where the contribution of each individual technique is proportional to its predictive accuracy. The performance of the models was assessed by randomly splitting data into 10 folds, and for each, using a random 70 % split dataset to calibrate the models and the remaining 30 % dataset to evaluate their predictive accuracy. Only individual models performing better than AUC > 0.8 and TSS > 0.7 were used to build each species ensemble model.

Lastly, ensemble models with evaluation metrics poorer than AUC < 0.8 and TSS < 0.7 were discarded [35].

3.2. Refining species distribution models

As an additional precautionary measure, we modified SDMs and ESMs to restrict predictions to areas geographically close to observed occurrences following the methods described in Thornhill et al. [36]. We adapted the parameters used to build the distance decay function to the characteristics of our study area (larger and usually with fewer occurrences per species) by using a sigma value of 500 km. We chose this value after calibration using values between 50 km and 5000 km. Five hundred km was considered the most suitable choice, especially because it was able to correct maps of species that are restricted to one slope of the Andean range (western or eastern), but typically have areas projected as suitable on the opposite slope where similar climatic conditions exist.

The incorporation of these model modifications has the potential to eliminate some areas where species are actually present (which can result in some underestimation of the

network performance). However, conservative approaches such as this are preferred in conservation research, where it is crucial to minimize false positive errors (e.g. considering that species are present in regions where they are not), which are especially harmful for conservation planning [37, 38].

3.3. Future scenarios

Future projections of species bioclimatic niches for year 2070 were built for two climate change scenarios from the IPCC fifth Assessment Report. Scenarios from this assessment are called representative concentration pathways (RCP) and correspond to specific pathways of accumulation of radiative forcing [39]. We evaluated RCP 4.5, a scenario of intermediate mitigation of greenhouse gases emissions in which there is a projected increase in average temperatures of 1.7 – 3.2°C by 2100, and RCP 8.5, a business-as-usual high emissions scenario with a larger projected increase of 3.2 – 5.4°C [40, 39]. Projections were calculated by averaging estimates from two global circulation models (GCMs): the Hadley Global Environment Model 2 (HadGEM2-ES) and the new Max Planck Institute Earth System Model (MPI-ESM-LR).

3.4. Dispersal restrictions in future scenarios

We applied restrictions to species dispersal in future scenarios. Maximum dispersal distances were different for the different taxa to acknowledge variability in their dispersal ability. For mammals, annual dispersal was calculated for each species as a function of their body mass and their feeding habit following equations in Schloss et al. [41]. Dispersal distances for projections in climate change scenarios have been less studied in other taxa, forcing us to use simpler rules. In the case of birds, we permitted a dispersal of 3 km/yr [42]. For plants, we allowed a dispersion of 1 km/yr [43]. We also used a 1 km/yr annual dispersal distance for amphibians and reptiles, taxa with restricted dispersal ability [44]. Each species SDMs or ESMs projections were truncated at the permitted dispersal distance.

Considering restrictions to species dispersal based on thresholds defined for each taxonomic group is not ideal but was preferred over unrealistic unlimited dispersal as a conservative approach.

3.5. Known limitations of species distribution models

As any model, SDMs are a simplification of the real world [14]. There are several uncertainties involving the projection of current and future distributions of species, involving biases in occurrence data and issues with downscaled environmental data, modelling techniques and projecting distributions in space and time [45, 14]. These uncertainties have been discussed elsewhere, and are addressed to some extent here [45, 46, 47].

In this context, it is possible that some of the biases in the occurrence data and environmental variables has been transferred to distribution models (but see above for mitigation). There are known problems in the region regarding Worldclim bioclimatic variables that arise from extrapolating across a restricted number of meteorological stations [48]. However, we have followed best practices at all steps of the modeling process in order to minimize the effect of these issues on our data, including: reduction of collinearity in environmental predictors, use of ensembles of three modeling techniques, ESMs, replication of bias in background data, use of two GCMs, distance corrections of models, and the inclusion of restrictions to dispersal. Moreover, despite these weaknesses, SDMs have potentially less commission errors (i.e. are predicted as occupied by the species where it is actually absent) than other distribution data, such as species ranges, and are therefore more suitable for a more conservative approach to conservation evaluations [49], especially in the case of tropical regions [50]. In the context of conservation, reducing false positives is important because they can lead to wasted surveillance effort, or concentration of management effort in inappropriate areas [51].

In summary, although issues with species distribution models are necessary to acknowledge, their characteristics have made them become an increasingly important tool in ecology and conservation biology [14]. When used with caution [38], they have great potential to biodiversity and conservation studies, especially at regions with poor species data, such as tropical Andes [49].

4. Set of analyzed species

Table A1. Number of species by taxonomic group and threat category [9] included in the analysis.

Taxa	Species	CR	EN	VU	NT	DD	LC	NE
Aves	1810	12	55	95	119	2	1368	159
Mammalia	197	7	11	20	10	19	127	3
Amphibia	699	28	79	72	48	58	375	39
Reptilia	452	1	3	8	7	5	94	334
Plants	13,352	8	98	306	175	18	290	12,457
Bryophytes	596	0	0	0	0	0	1	595
Ferns and allies	902	0	2	8	4	0	7	881
Flowering plants	11,836	8	95	295	169	18	271	10,980
Gymnosperms	18	0	1	3	2	0	11	1
**Total**	**16,510**	**56**	**246**	**501**	**359**	**102**	**2254**	**12,993**

CR: Critically endangered; EN: Endangered; VU: Vulnerable; NT: Near Threatened; DD: Data Deficient; LC: Least Concern; NE: Not Evaluated

5. Randomization analysis – Expanded details

5.1. Framework and algorithm description

We produced eight randomization tests, one for each of the evaluated periods (1937-1945, 1946-1955, 1956-1965, 1966-1975, 1976-1985, 1986-1995, 1996-2005, and 2006-2015). For each test, we created 100 alternative random networks (hereafter, 'random (PA) networks'), in which a randomization algorithm was used to find an alternative random location for each PA declared during the period.

Our randomization algorithm was inspired by Model II in Rodrigues et al. [52] and is equivalent to the algorithm used in Vos & Cumming [53]. It was implemented in R [22] and uses the function 'elide' from the 'maptools' library [54]. Specifically, the algorithm repeats the following routine for each PA declared in a given evaluated period:

1. It finds a new random location for the PA polygon by (1) selecting a new random point within the limits of the study area, which is used as the new centroid for the polygon, (2) rotating PA polygon by a value between 0º and 364º, and (3) randomly choosing to flip the polygon and use its mirror image or not.

2. It evaluates whether random new locations meet three constraints: they do not overlap with (1) the polygons of PAs declared in previous decades (at their actual location), with (2) polygons of PA from the same decade already randomized by the algorithm at a previous step, or with (3) the external border of the study area (without considering countries' internal frontiers).

3. A new random location for a PA is accepted if the three constraints are met, and the algorithm repeats the process for the next PA. If a random location is not accepted, the algorithm repeats (1) and (2) until an accepted location is found before moving to the next PA.

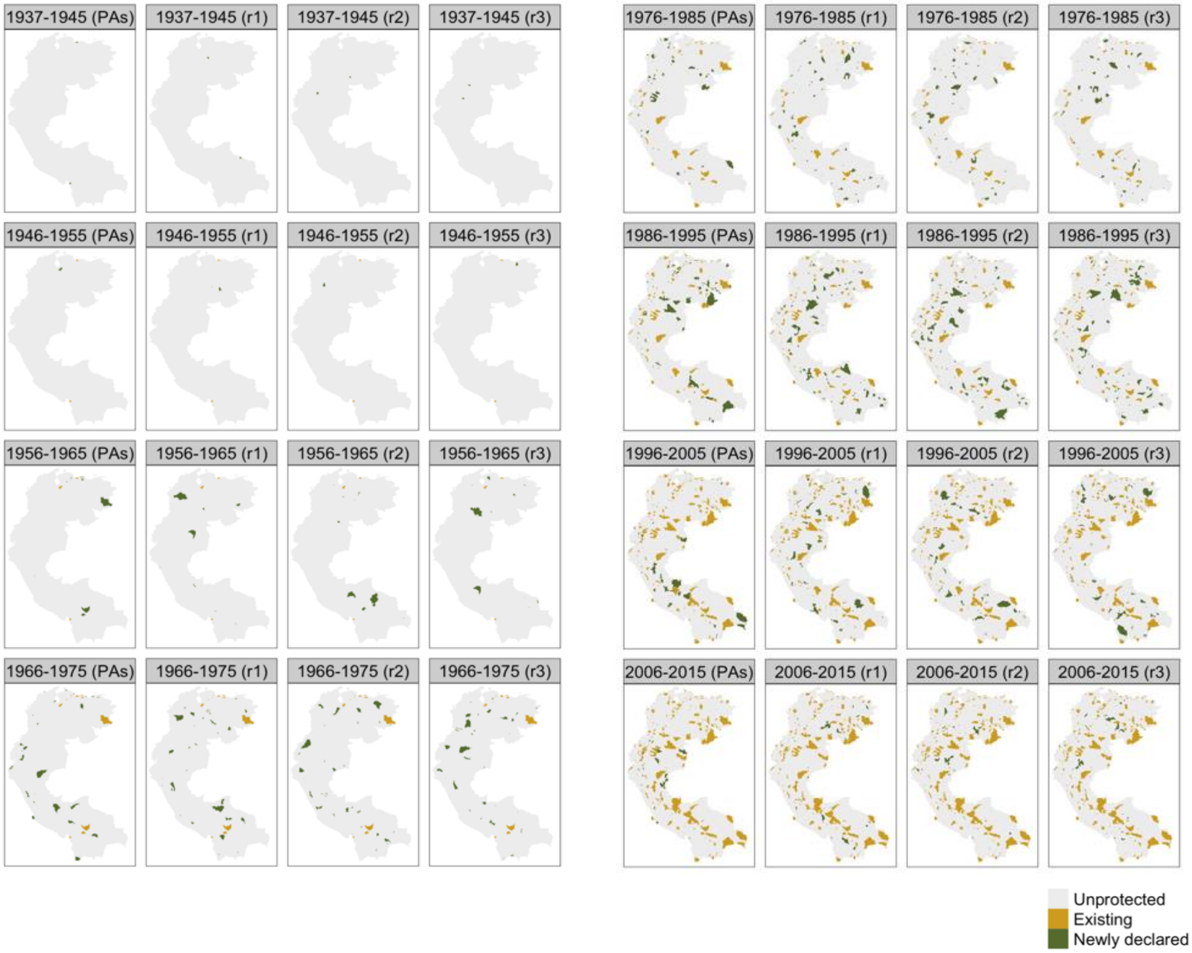

Figure A.4: Randomization analysis Framework. Maps show existing and newly declared PAs in the eight evaluated periods (left column: 1937-1945, 1946-1955, 1956-1965, 1966-1975; right column: 1976-1985, 1986-1995, 1996-2005, 2006-2015). Within each decade-period, the left-hand map (PAs) shows the actual location of PAs and the following maps show the first three random networks (r1, r2 and r3) resulting from applying the algorithm. Random networks are formed by existing PAs (yellow) at their actual location, and PAs declared during the decade (green) at random positions. Figure created in R v4.0.0 (https://cran.r-project.org/).

5.2. Framework results

Each random network produced is formed by the same catalogue of individual PA polygons than actual PA networks, and thus they preserve a few important properties: they have the same (1) total surface-area protected, (2) number of PA units, and (3) distribution of PA sizes (Fig. A.4). Other PA network properties such as PA shape and perimeter are also preserved. These PA properties are known to influence PA performance [55] and management cost [56, 57]. For instance, large PAs tend to have reduced management costs because they require patrolling a smaller perimeter due to a reduced perimeter/area ratio [55].

Constraints used to ensure constant area protected and distributions of PA size and number resulted in a semi-random selection of land across 100 repetitions considering a 1km grid (Fig. A.5). Pixels in the grid that are distant from the study area border and previously existing PAs tend to have large selection frequencies because the random placement of both large and small PA polygons is likely to be accepted due to non-overlaps. In contrast, areas near the study-area boundary and previously existing PAs can only accommodate small PAs without incurring in non-allowed overlaps, and thus these areas tend to result in smaller selection frequency.

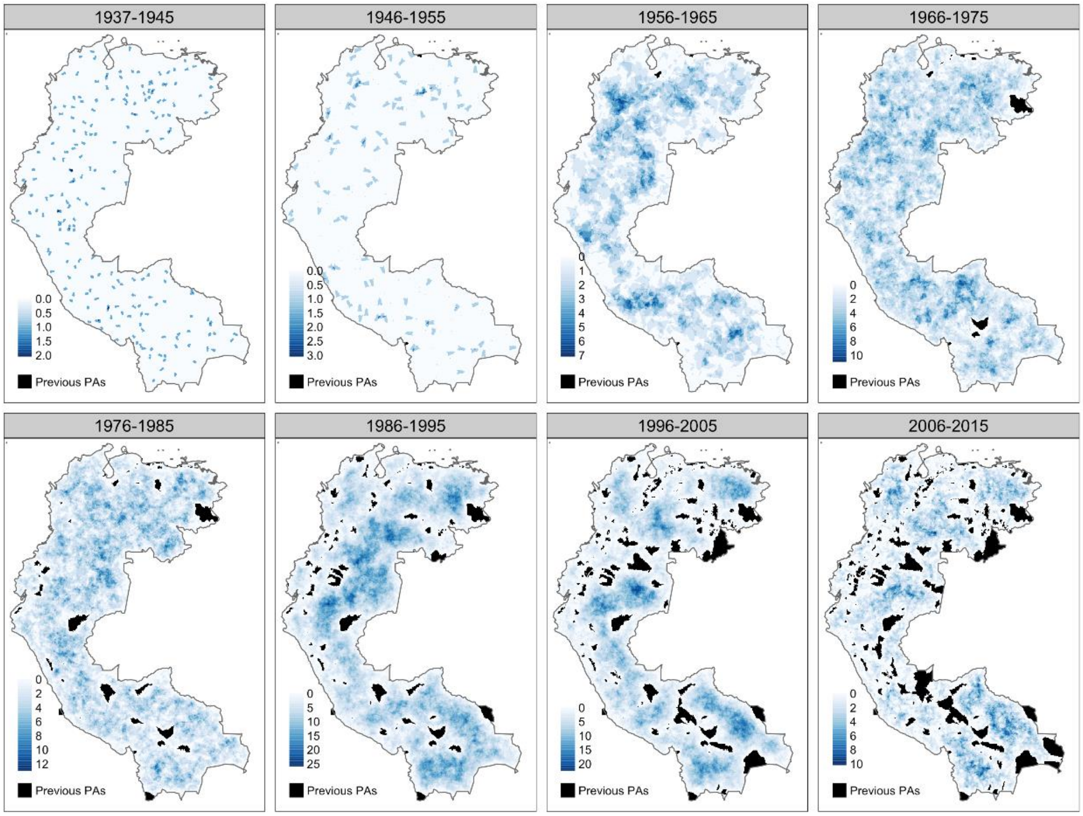

Figure A.5: Selection frequency across random networks and evaluated periods. The color scale shows selection frequency on a 1km grid across the 100 random networks in each randomization test, with darker blue indicating cells with higher selection. PAs declared in periods older than the period under evaluation (black) maintain their actual location. Figure created in R v4.0.0 (https://cran.r-project.org/).

Heterogeneous selection frequency is, however, an expected outcome of the randomization approach, and it is coherent with real-world situations. Decision-makers will only have the option to introduce small new PAs in regions where only small patches of unprotected landscape remain available for selection. Contrastingly, they may have the choice of creating either one large PA or several small PAs in the case of large regions without previously existing PAs. However, there are differences with real-world scenarios because our

framework does not allow for PA polygons to adapt their shapes to fit other PAs or study area borders naturally, which may prevent some network configurations. Future randomization algorithms may explore making PA shapes adaptable to other reserves and political or natural borders.

5.3. Sensitivity analysis

We tested the sensitivity of the randomization analysis by considering a range of species representation targets. Specifically, we explored the following cases which present variations of representation targets:

- Targets scaled by species' range size: based on the same method described for the main analysis, in which species' targets are log-linearly scaled between an upper and lower thresholds of range size. We assessed the following variations of targets: (1) lower overall targets, with values scaled between 70% of distributions of species with a modelled range of 1000 km^2 or smaller and 10% for those with ranges of 55,000 km^2 or larger; (2) higher targets, especially for range-restricted species, with values scaled between 100% for species with a modelled range of 500 km^2 or smaller and 20% for those with ranges of 100,000 km^2 or larger.
- Fixed targets for all species: based on a method where the same target is used for all species. We assessed the following fixed values (3) 10% of species modelled range; (4) 17%; (5) 20%; (6) 30%; (7) 40%. The 17% representation target was included because this value is frequently used in the literature due its connection to the PA-network ecological representativeness Aichi Biodiversity Target 11 [58].

We found that the main result is robust to target setting variations. Most variations rendered results equivalent to those presented in the main results (variations 1, and 4-6), in which the observed representation performance was not significantly larger than that of random networks for old decades but becomes significantly larger in recent decades of PA expansion (since 1976-1985 or 1986-1995).

Some variations did not show the same trend of better performance than random networks in recent decades (variations 2, 3 and 7). These variations are characterized by representation targets that are either very small or vary high compared with the targets used in the main analysis. When targets are set too small (variation 1), most species meet their representation target by 1975-1995, which results in a minimal number of species left to be included in later decades. Similarly, setting too high targets (variations 2 and 7) builds a scenario where random and existing PAs are not able to fulfill the targets.

6. Patterns of under-represented species with decadal PA expansions

To aid the interpretation of the randomization analysis results, we produced maps showing PAs declared each decade overlaid with the richness of under-represented species (i.e., those with a representation target that is not met by PA coverage). We created the richness maps by counting the number of species not meeting their representation target in the PA network composed by areas from older decades, averaged by ecoregion to enhance contrast [59]. Results are provided for all species (Fig. A.6), threatened species (Fig. A.7), and separated taxa (Fig. A.8 to A.16).

In general, we found that efficient PAs representing species (i.e., more efficient than expected at random) were seen for decades where PA expansions concentrated over ecoregions with a high richness of under-represented species, such as low elevations in the Amazon and Andean foothills (i.e., the last decade).

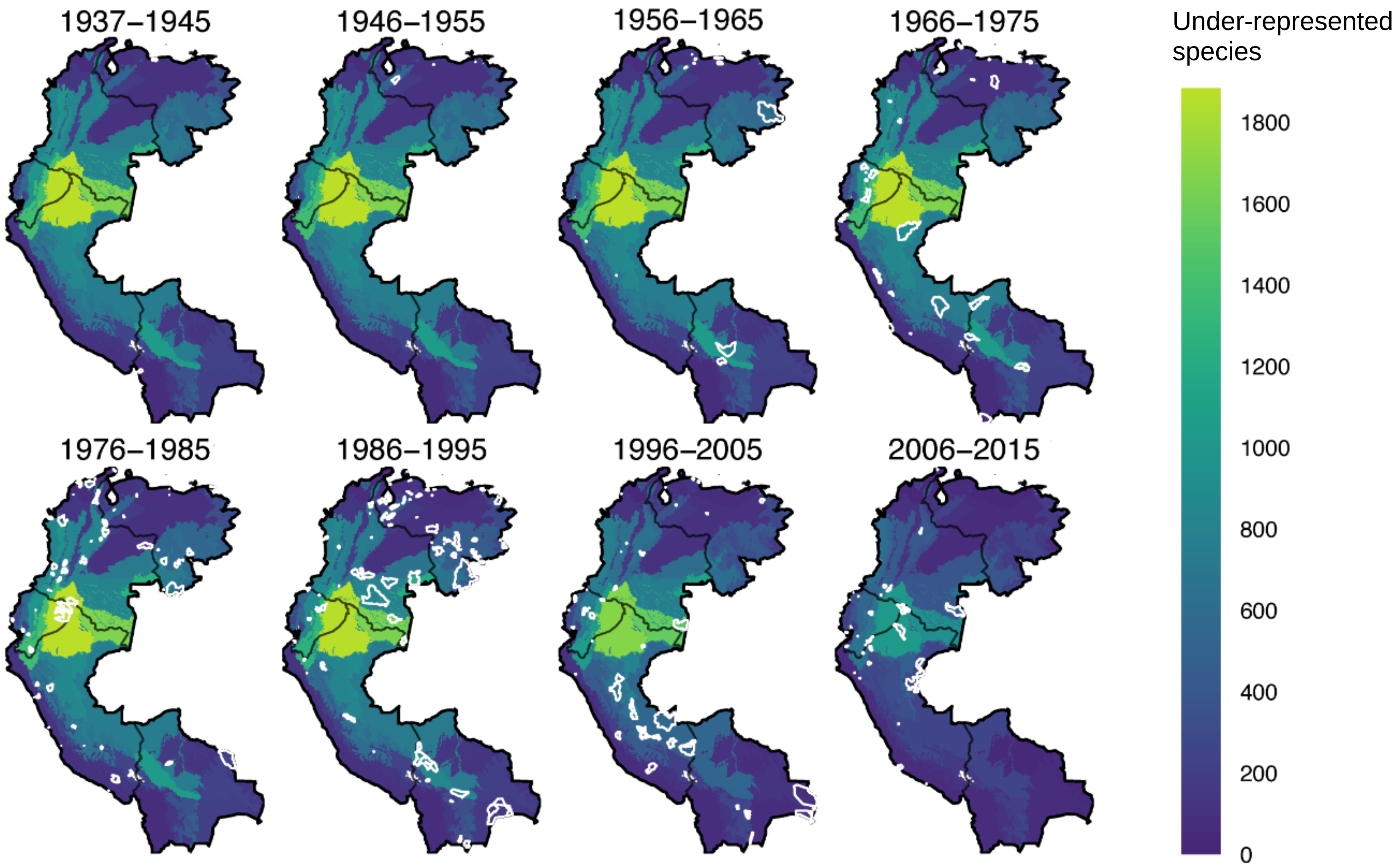

Figure A.6. Location of PAs declared each decade (outlined in white) over richness of under-represented species (i.e., number of species per ecoregion not meeting target in the PA network previous to expansion) for **all species**. Figure created in R v4.0.0 (https://cran.r-project.org/).

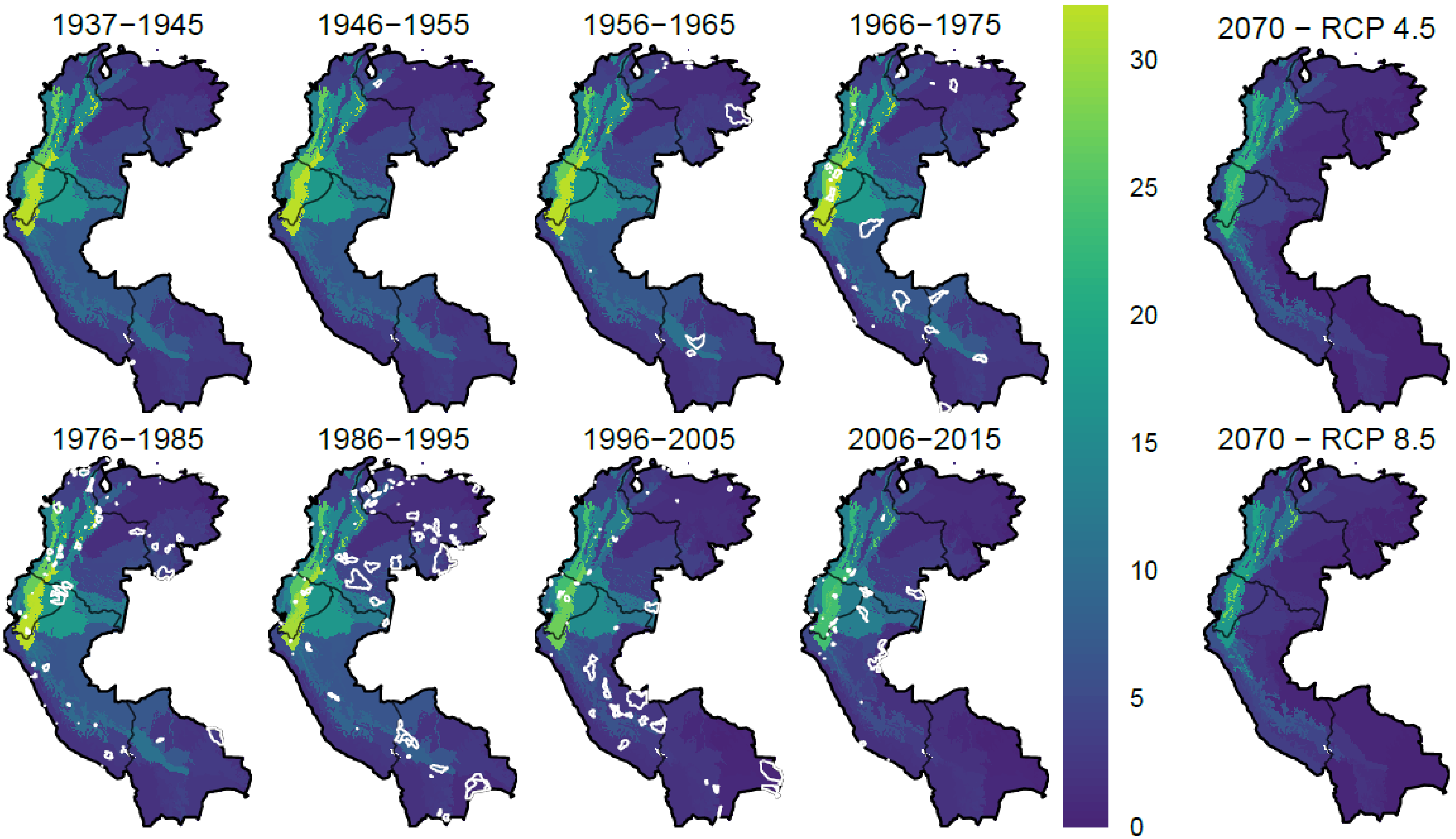

Figure A.7. Location of PAs declared each decade (outlined in white) over richness of under-represented **threatened** species. Figure created in R v4.0.0 (https://cran.r-project.org/).

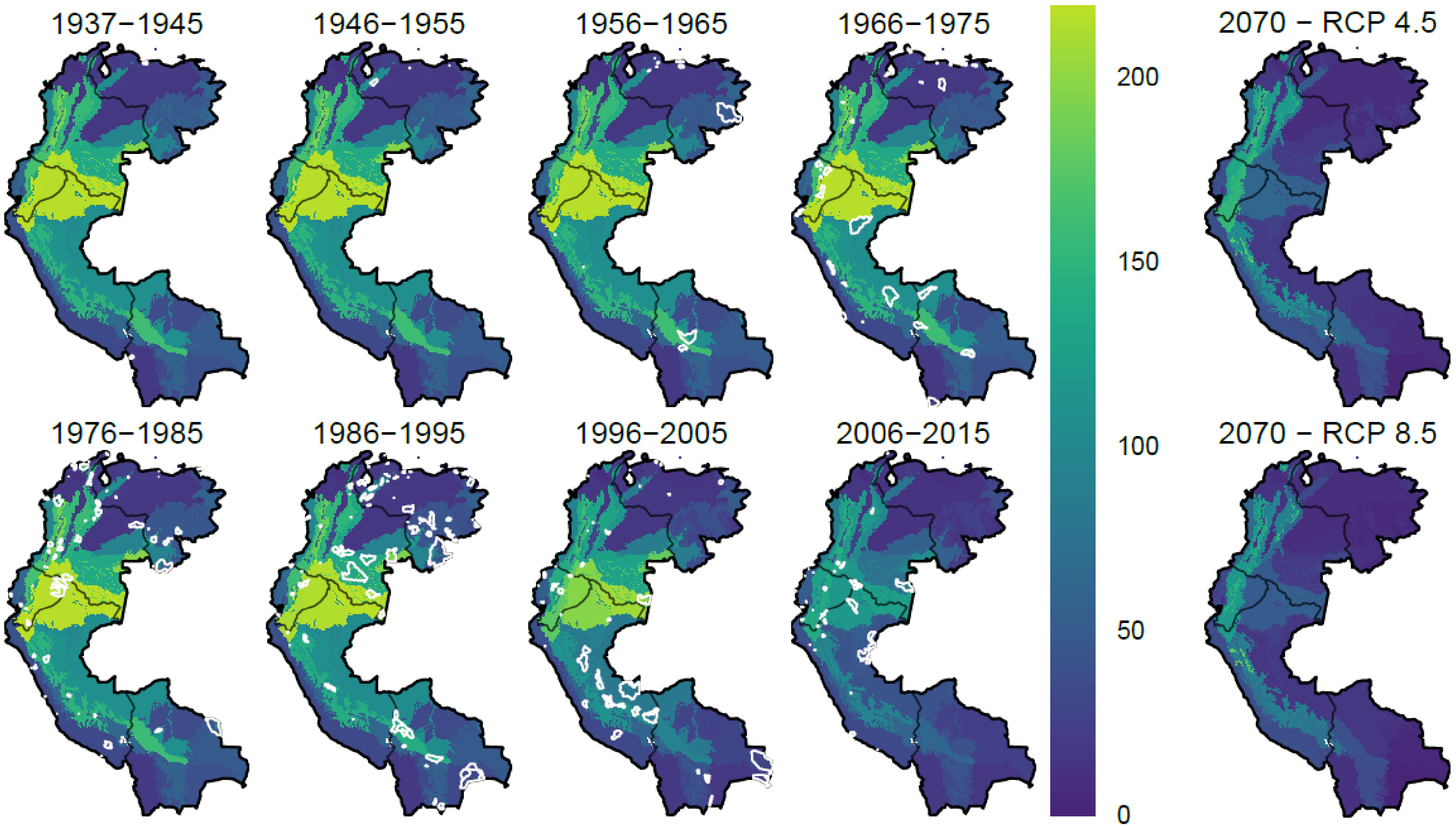

Figure A.8. Location of PAs declared each decade (outlined in white) over richness of under-represented species for **birds**. Figure created in R v4.0.0 (https://cran.r-project.org/).

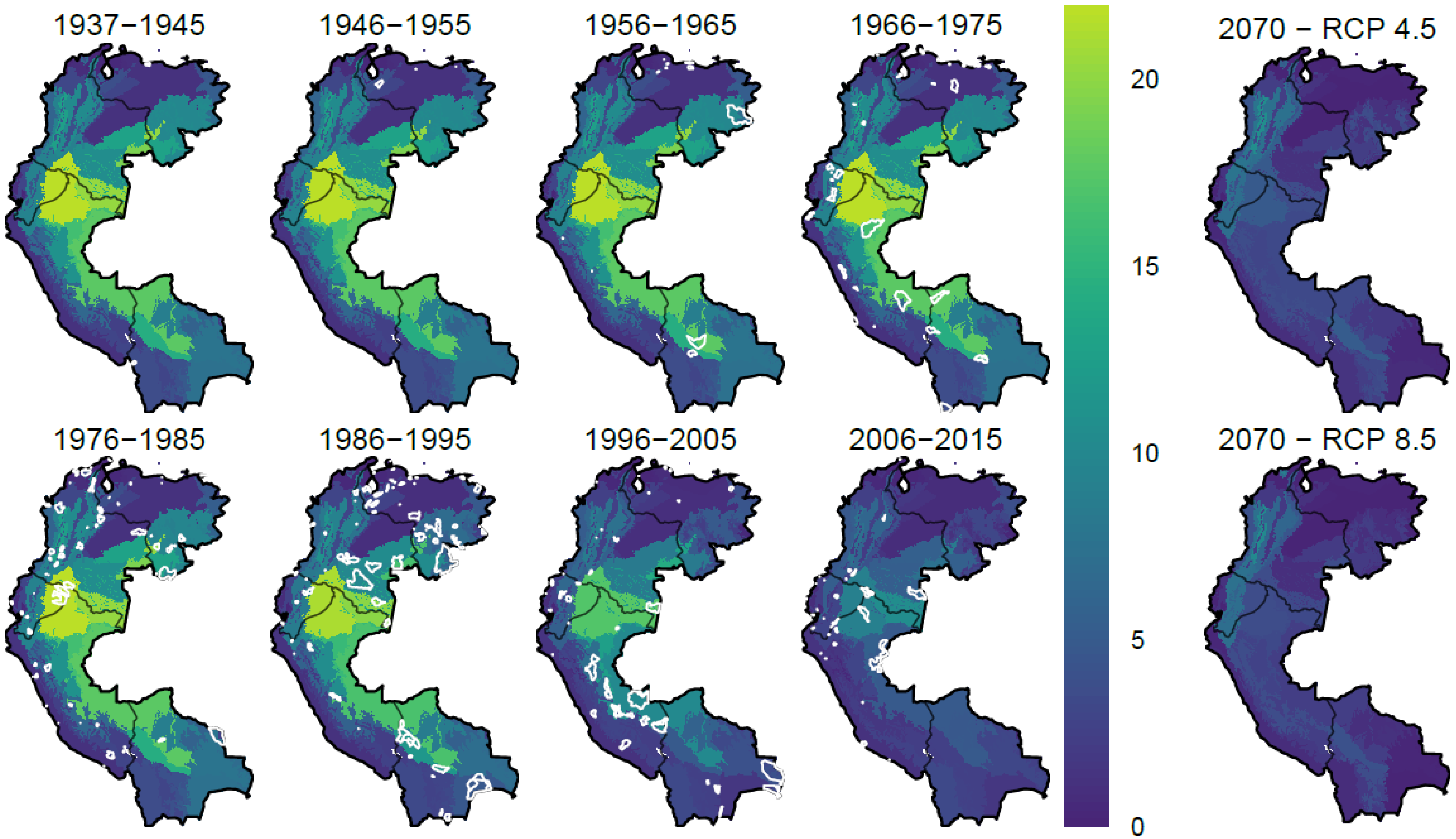

Figure A.9. Location of PAs declared each decade (outlined in white) over richness of under-represented species for **mammals**. Figure created in R v4.0.0 (https://cran.r-project.org/).

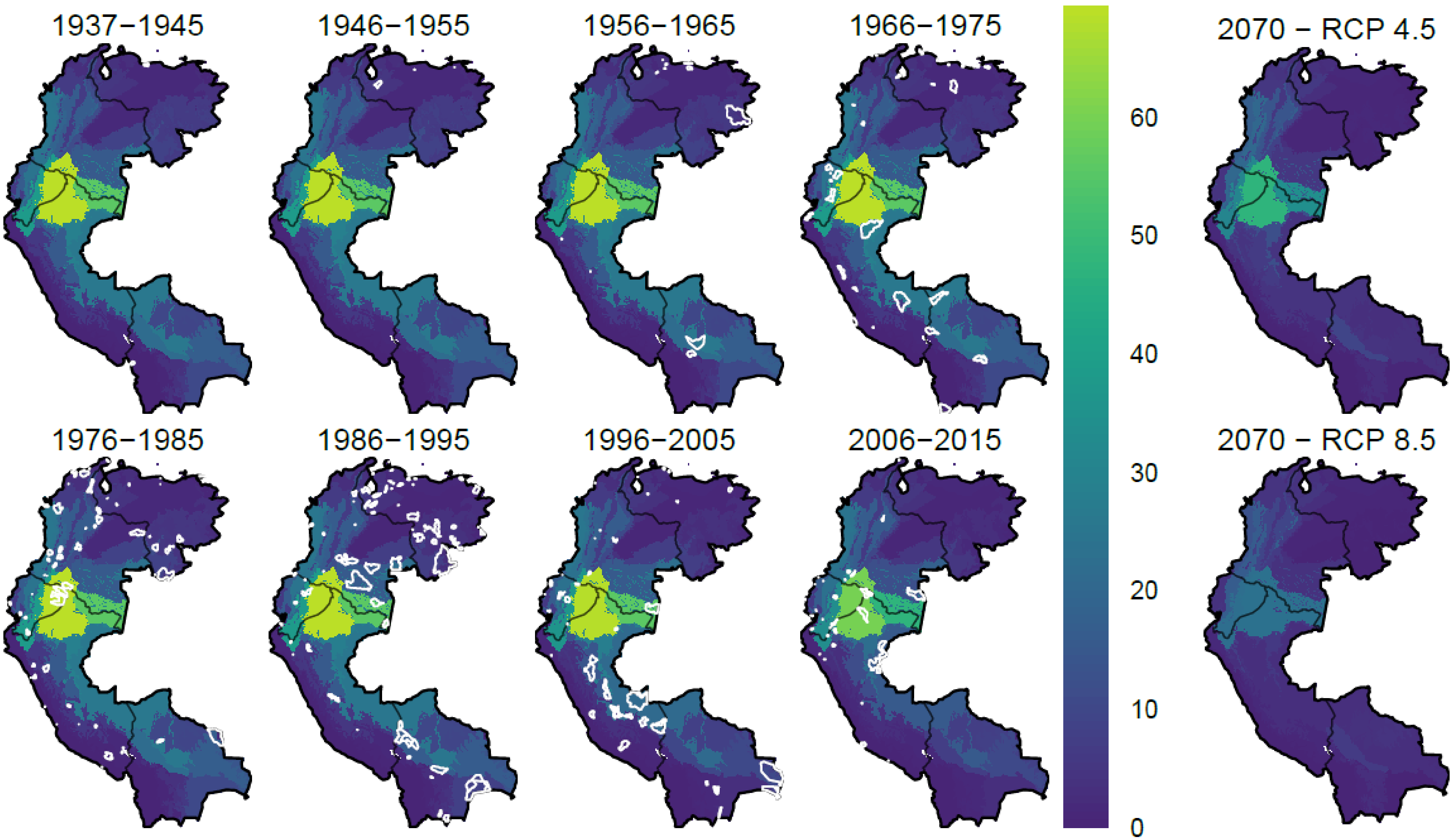

Figure A.10. Location of PAs declared each decade (outlined in white) over richness of under-represented species for **amphibians**. Figure created in R v4.0.0 (https://cran.r-project.org/).

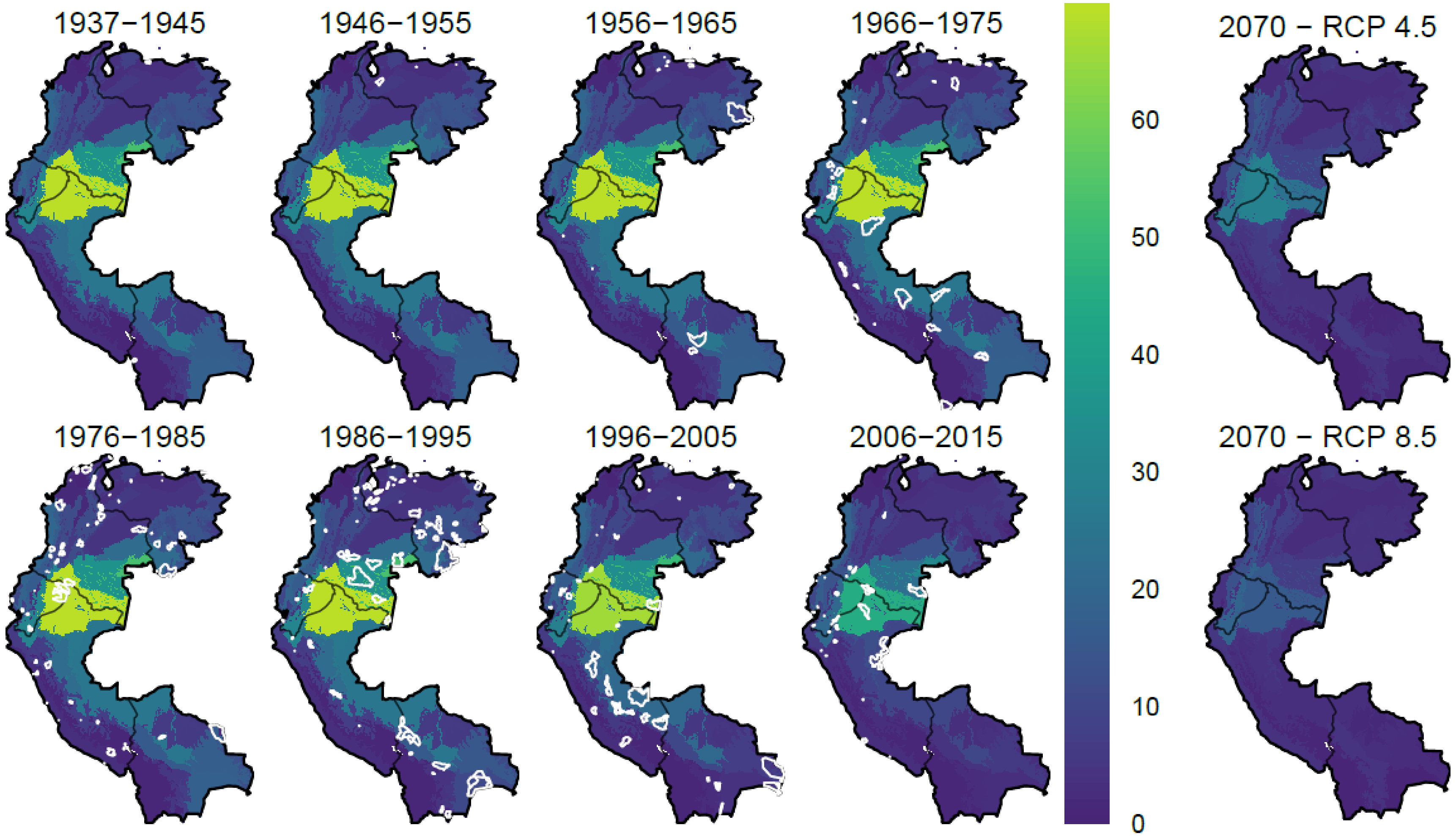

Figure A.11. Location of PAs declared each decade (outlined in white) over richness of under-represented species for **reptiles**. Figure created in R v4.0.0 (https://cran.r-project.org/).

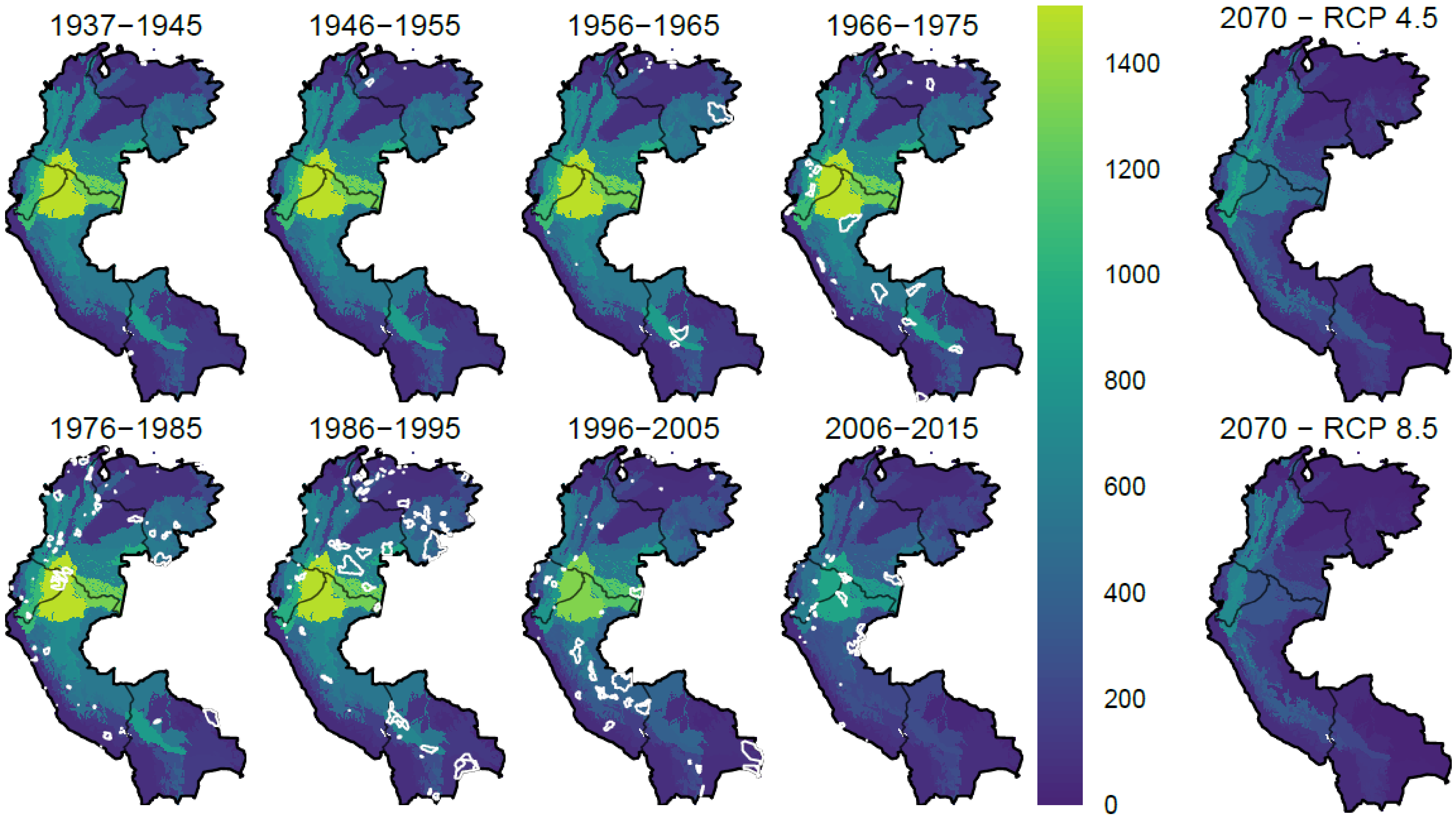

Figure A.12. Location of PAs declared each decade over richness of under-represented species for **plants** (including gymnosperms, flowering plants, bryophytes, ferns and allies). Figure created in R v4.0.0 (https://cran.r-project.org/).

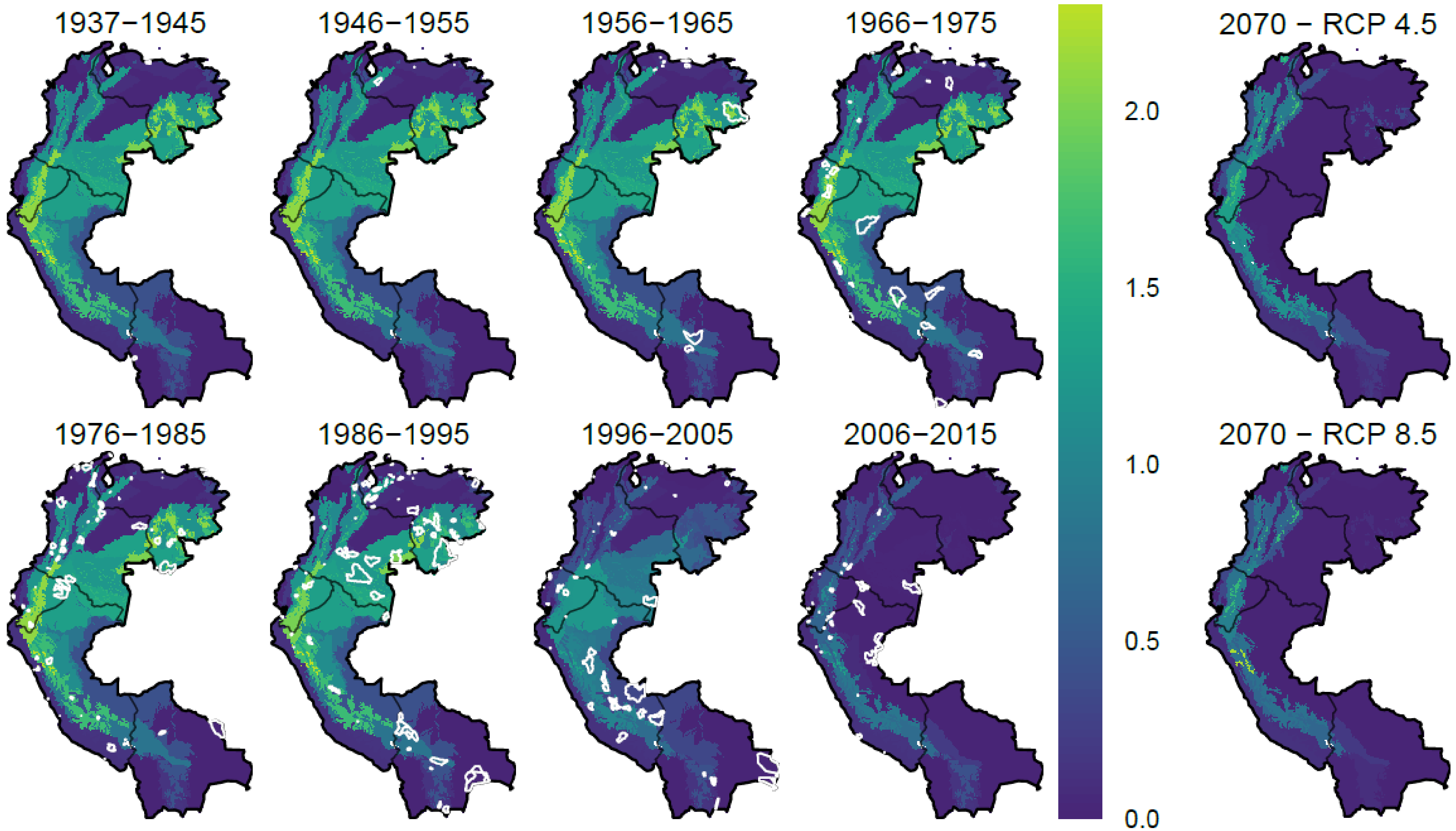

Figure A.13. Location of PAs declared each decade (outlined in white) over richness of under-represented species for **gymnosperms**. Figure created in R v4.0.0 (https://cran.r-project.org/).

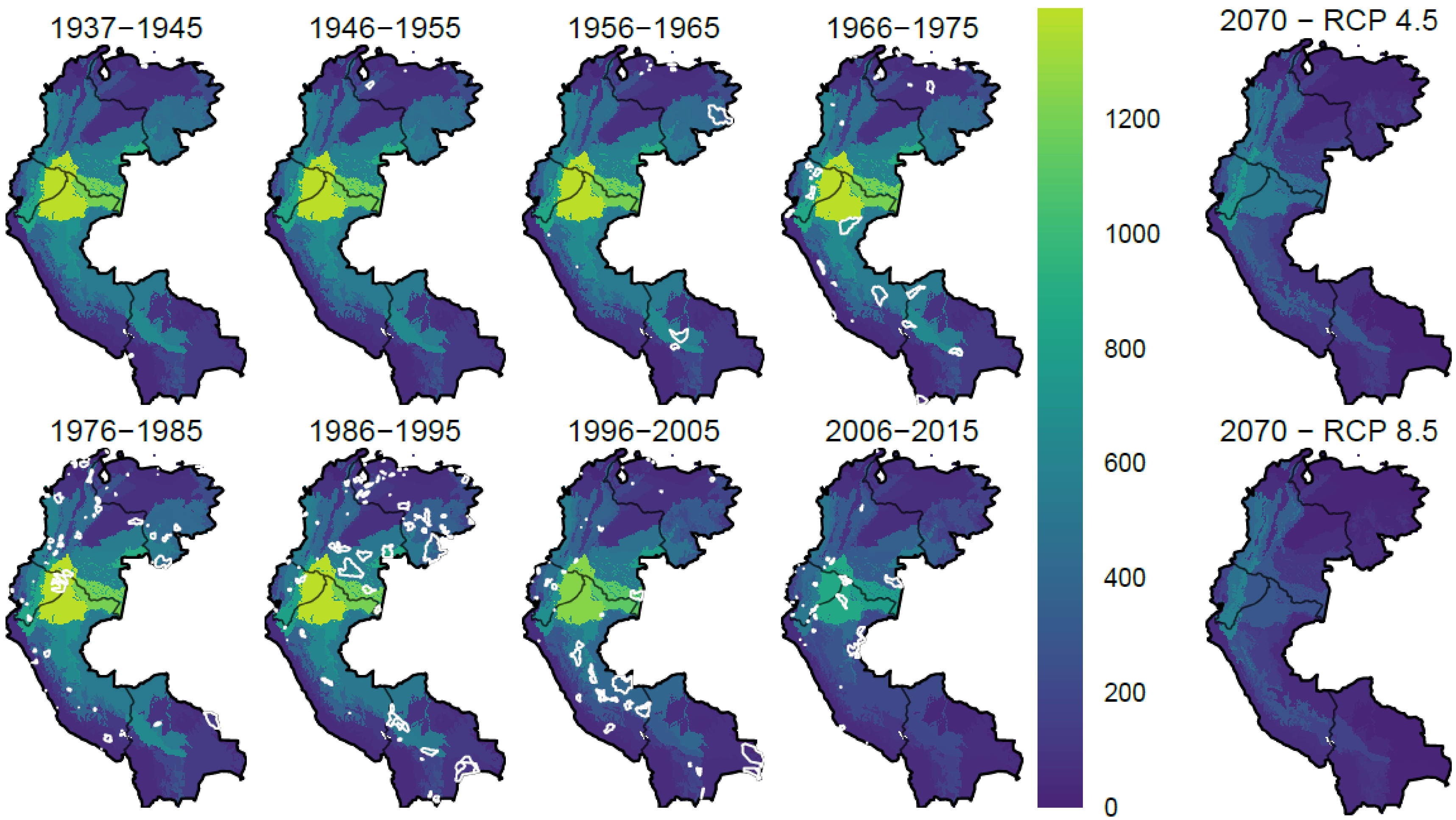

Figure A.14. Location of PAs declared each decade (outlined in white) over richness of under-represented species for **flowering plants**. Figure created in R v4.0.0 (https://cran.r-project.org/).

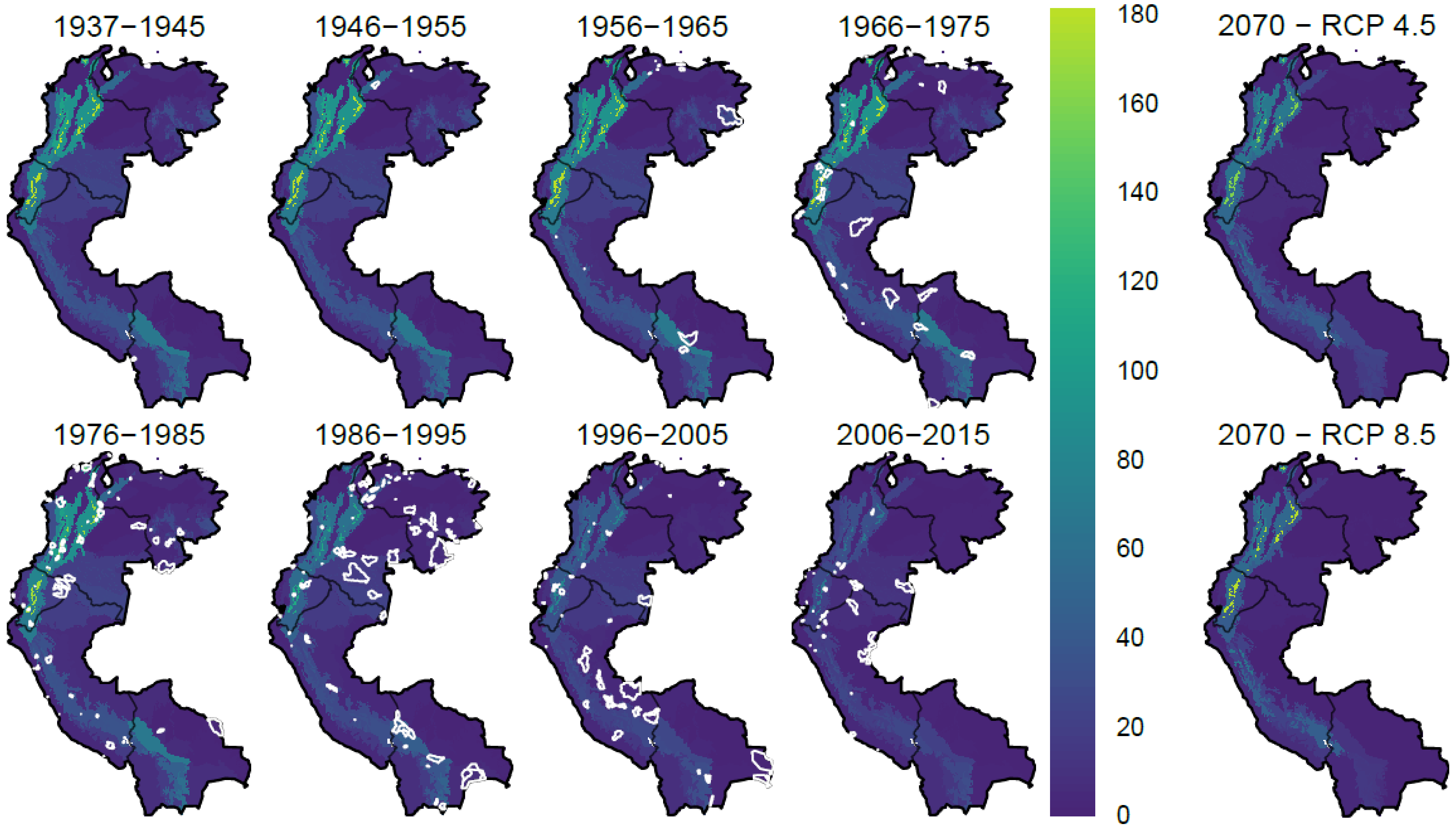

Figure A.15. Location of PAs declared each decade (outlined in white) over richness of under-represented species for **bryophytes**. Figure created in R v4.0.0 (https://cran.r-project.org/).

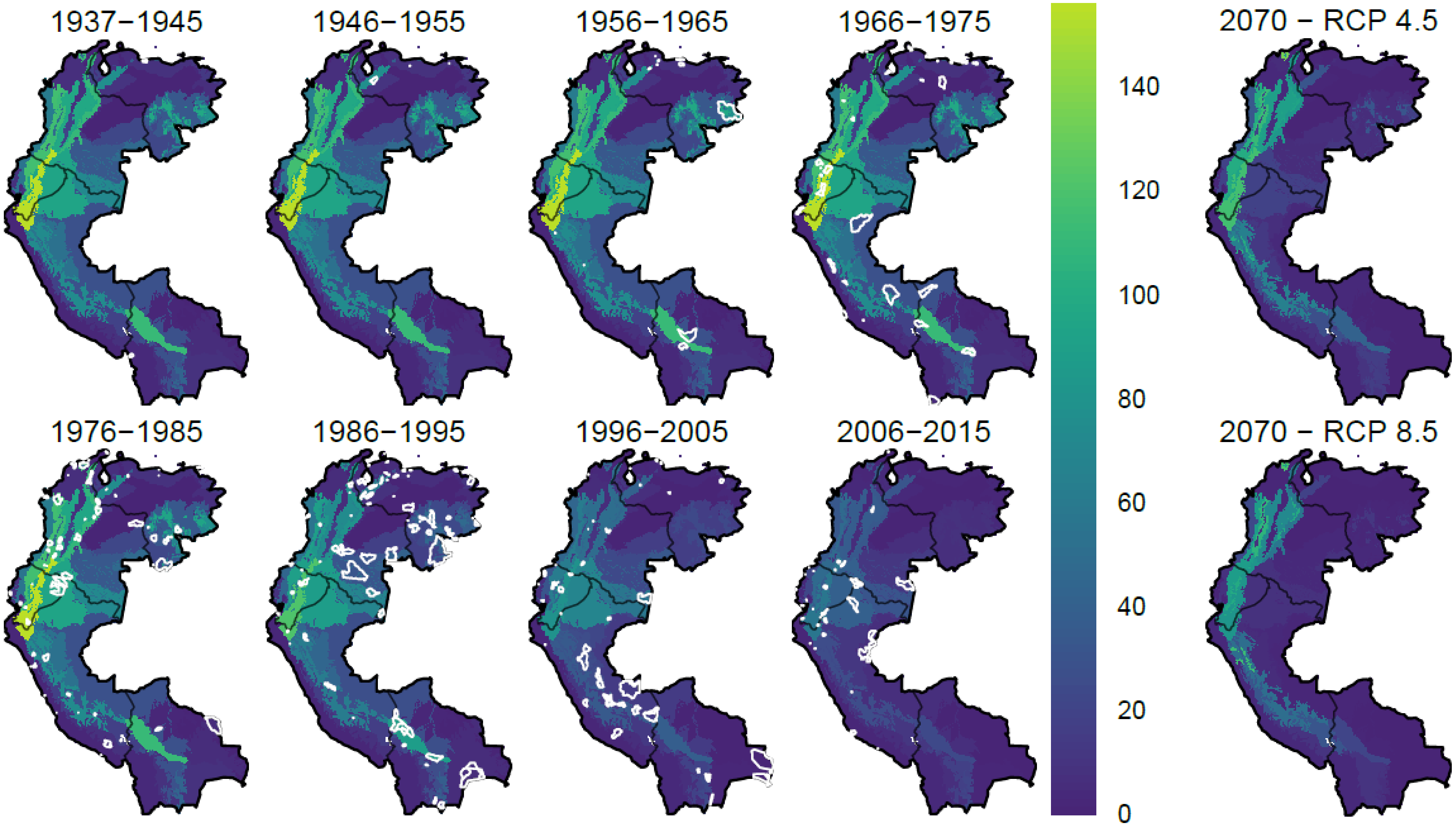

Figure A.16. Location of PAs declared each decade (outlined in white) over richness of under-represented species for **ferns and allies**. Figure created in R v4.0.0 (https://cran.r-project.org/).

8. References

1. INPARQUES (1991) Decreto de Creación Monumentos Naturales "Tepuyes". In, p. 9. Ministerio del Poder Popular para el Ambiente Caracas.

2. García, Rafael, and María Silva. "Las ABRAE versus las áreas protegidas en Venezuela." COPÉRNICO Revista arbitrada de divulgación científicaCOPÉRNICO Revista arbitrada de divulgación científica Dec (2014): 27–29.

3. Frost D.R. (2014) Amphibian Species of the World: an Online Reference. Version 6.0. Electronic Database accessible at http://research.amnh.org/herpetology/amphibia/index.html. American Museum of Natural History, New York, USA.

4. Remsen J.V., Areta J.I., Cadena C.D., Claramunt S., Jaramillo A., Pacheco J.F., . . . Zimmer K.J. A classification of the bird species of South America. American Ornithologists' Union. Version April 2014. http://www.museum.lsu.edu/~Remsen/SACCBaseline.htm.

5. IUCN. (2014) The IUCN Red List of Threatened Species. Version 2014.1. http://www.iucnredlist.org. Downloaded on January 2015.

6. Uetz P., Freed P., Hošek J. The Reptile Database, http://www.reptile-database.org, accessed April 2014.

7. Enquist B.J., Condit R., Peet R.K., Schildhauer M., Thiers B. (2009) The Botanical Information and Ecology Network (BIEN): Cyberinfrastructure for an integrated botanical information network to investigate the ecological impacts of global climate change on plant biodiversity. PeerJ Preprints (No. e2615v1). doi: 10.7287/peerj.preprints.2615v2

8. Maitner B., Boyle B., Casler N., Condit R., Donoghe J.C., Duran S.M., . . . Enquist B.J. (2017) The BIEN R package: A tool to access the Botanical Information and Ecology Network (BIEN) Database.

9. IUCN. (2017) The IUCN Red List of Threatened Species. Version 2017-1. Accessed in April 2017.

10. Chamberlain S., Bartomeus I. (2016) originr: Fetch Species Origin Data from the Web.

11. Chamberlain S., Szocs E., Boettiger C., Ram K., Bartomeus I., Baumgartner J., . . . O'Donnell J. (2016) taxize: Taxonomic information from around the web.

12. Wallace R., López-Strauss H., Mercado N., Porcel Z. (2013) Base de datos sobre la distribución de los mamíferos medianos y grandes de Bolivia. Wildlife Conservation Society, La Paz.

13. Duellman W.E., Lehr E. (2009) Terrestrial-Breeding Frongs (Strabomantidae) in Peru. Natur und Tier-Verlag Gmb, Münster.

14. Guisan A., Thuiller W., Zimmermann N.E. (2017) Habitat Suitability and Distribution Models: With Applications in R. Cambridge University Press, Cambridge.

15. Maldonado C., Molina C.I., Zizka A., Persson C., Taylor C.M., Albán J., . . . Antonelli A. (2015) Estimating species diversity and distribution in the era of Big Data: To what extent can we trust public databases? Global Ecology and Biogeography **24**, 973-984. doi: 10.1111/geb.12326

16. BirdLife International, NatureServe. (2015) Bird species distribution maps of the world. Version 5.0. BirdLife International, Cambridge, UK and NatureServe, Arlington, USA.

17. IUCN. (2017) The IUCN Red List of Threatened Species. Version 2017-1. Accessed in April 2017.

18. Meyer C., Weigelt P., Kreft H. (2016) Multidimensional biases, gaps and uncertainties in global plant occurrence information. Ecology Letters, 992-1006. doi: 10.1111/ele.12624

19. Schulman L., Ruokolainen K., Junikka L., Sääksjärvi I.E., Salo M., Juvonen S.K., . . . Higgins M. (2007) Amazonian biodiversity and protected areas: Do they meet? Biodiversity and Conservation **16**, 3011-3051. doi: 10.1007/s10531-007-9158-6

20. Guisan A., Zimmermann N.E. (2000) Predictive habitat distribution models in ecology. Ecological Modelling **135**, 147-186. doi: https://doi.org/10.1016/S0304-3800(00)00354-9

21. Thuiller A.W., Georges D., Engler R., Georges M.D., Thuiller C.W. (2013) Package 'biomod2'.

22. R Core Team. (2017) R: A language and environment for statistical computing. R Foundation for Statistical Computing, Vienna, Austria.

23. Phillips S.J., Anderson R.P., Schapire R.E. (2006) Maximum entropy modeling of species geographic distributions. Ecological Modelling **190**, 231-259. doi: https://doi.org/10.1016/j.ecolmodel.2005.03.026

24. Breiman L. (2001) Random forests. Machine Learning **45**, 5-32. doi: 10.1023/A:1010933404324

25. Friedman J.H. (2001) Greedy Function Approximation : A Gradient Boosting Machine. The Annals of Statistics **29**, 1189-1232.

26. Hijmans R.J., Cameron S.E., Parra J.L., Jones P.G., Jarvis A. (2005) Very high resolution interpolated climate surfaces for global land areas. International Journal of Climatology **25**, 1965-1978. doi: https://doi.org/10.1002/joc.1276

27. Booth, Trevor H. (2022) Checking Bioclimatic Variables That Combine Temperature and Precipitation Data before Their Use in Species Distribution Models. Austral Ecology 47, 7: 1506–14. https://doi.org/10.1111/aec.13234.

28. Mateo R.G., Broennimann O., Petitpierre B., Muñoz J., van Rooy J., Laenen B., . . . Vanderpoorten A. (2015) What is the potential of spread in invasive bryophytes? Ecography **38**, 480-487. doi: 10.1111/ecog.01014

29. Phillips S.J., Dudík M., Elith J., Graham C.H., Lehmann A., Leathwick J., Ferrier S. (2009) Sample selection bias and presence-only distribution models : implications for background and pseudo-absence data Reference Sample selection bias and presence-only distribution models: implications for background and pseudo-absence data. Ecological Applications **19**, 181-197. doi: 10.1890/07-2153.1

30. Cayuela, L., Golicher, D. J., Newton, A. C., Kolb, M., de Alburquerque, F. S., Arets, E. J. M. M., Alkemade, J. R. M., and Pérez, A. M. (2009) Species Distribution Modeling in the Tropics: Problems, Potentialities, and the Role of Biological Data for Effective

Species Conservation. Tropical Conservation Science 2:3. 319–52. https://doi.org/10.1177/194008290900200304.

31. Venter O., Sanderson E.W., Magrach A., Allan J.R., Beher J., Jones K.R., . . . Watson J.E.M. (2016) Sixteen years of change in the global terrestrial human footprint and implications for biodiversity conservation. Nature Communications **7**, 12558. doi: 10.1038/ncomms12558

32. Phillips, S. J., Dudík, M., Elith, J., Graham, C. H., Lehmann, A., Leathwick, J., and Ferrier, S. (2009) Sample Selection Bias and Presence-Only Distribution Models: Implications for Background and Pseudo-Absence Data. Ecological Applications 19:1, 181–97. https://doi.org/10.1890/07-2153.1.

33. van Proosdij A.S.J., Sosef M.S.M., Wieringa J.J., Raes N. (2015) Minimum required number of specimen records to develop accurate species distribution models. Ecography **39**, 542-552. doi: 10.1111/ecog.01509

34. Breiner F.T., Guisan A., Bergamini A., Nobis M.P. (2015) Overcoming limitations of modelling rare species by using ensembles of small models. Methods in Ecology and Evolution **6**, 1210-1218. doi: 10.1111/2041-210X.12403

35. Collevatti R.G., Terribile L.C., Lima-Ribeiro M.S., Nabout J.C., Oliveira G.d., Rangel T.F., . . . Diniz-Filho J.a.F. (2012) A coupled phylogeographical and species distribution modelling approach recovers the demographical history of a Neotropical seasonally dry forest tree species. Molecular Ecology **21**, 5845-5863. doi: 10.1111/mec.12071

36. Thornhill A.H., Baldwin B.G., Freyman W.A., Nosratinia S., Kling M.M., Morueta-holme N., . . . Mishler B.D. (2017) Spatial phylogenetics of the native California flora. BMC Biology **15**, 96. doi: 10.1186/s12915-017-0435-x

37. Baxter P.W.J., Possingham H.P. (2011) Optimizing search strategies for invasive pests: Learn before you leap. Journal of Applied Ecology **48**, 86-95. doi: 10.1111/j.1365-2664.2010.01893.x

38. Loiselle B., Howell C.A., Graham C.H., Goerck J.M., Brooks T., Smith K.G., Williams P.H. (2003) Avoiding pitfalls of using species distributions models in conservation

planning. Conservation Biology **17**, 1591-1600. doi: https://doi.org/10.1111/j.1523-1739.2003.00233.x

39. Moss R.H., Edmonds J.A., Hibbard K.A., Manning M.R., Rose S.K., Van Vuuren D.P., . . . Wilbanks T.J. (2010) The next generation of scenarios for climate change research and assessment. Nature **463**, 747-756. doi: 10.1038/nature08823

40. Fuss S., Canadell J.G., Peters G.P., Tavoni M., Andrew R.M., Ciais P., . . . Yamagata Y. (2014) Betting on negative emissions. Nature Climate Change **4**, 850-853. doi: 10.1038/nclimate2392

41. Schloss C.A., Nunez T.A., Lawler J.J. (2012) Dispersal will limit ability of mammals to track climate change in the Western Hemisphere. Proceedings of the National Academy of Sciences **109**, 8606-8611. doi: 10.1073/pnas.1116791109

42. Reside A.E., VanDerWal J., Kutt A.S. (2012) Projected changes in distributions of Australian tropical savanna birds under climate change using three dispersal scenarios. Ecology and Evolution **2**, 705-718. doi: 10.1002/ece3.197

43. Broennimann O., Thuiller W., Hughes G., Midgley G.F., Alkemade J.M.R., Guisan A. (2006) Do geographic distribution, niche property and life form explain plants' vulnerability to global change? Global Change Biology **12**, 1079-1093. doi: 10.1111/j.1365-2486.2006.01157.x

44. Gibbons J.W., Scott D.E., Ryan T.J., Buhlmann K.A., Tuberville T.D., Metts B.S., . . . Winne C.T. (2000) The Global Decline of Reptiles, Déjà Vu Amphibians: Reptile species are declining on a global scale. Six significant threats to reptile populations are habitat loss and degradation, introduced invasive species, environmental pollution, disease, unsustainable use, and global climate change. BioScience **50**, 653-666. doi: https://doi.org/10.1641/0006-3568(2000)050[0653:TGDORD]2.0.CO;2

45. Mateo R.G., Felicísimo Á.M., Muñoz J. (2011) Species distributions models: A synthetic revision. Revista Chilena de Historia Natural **84**, 217-240. doi: 10.4067/S0716-078X2011000200008

46. Segurado P., Araújo M.B. (2004) An evaluation of methods for modelling species distributions. Journal of Biogeography **31**, 1555-1568. doi: https://doi.org/10.1111/j.1365-2699.2004.01076.x

47. Thuiller W. (2004) Patterns and uncertainties of species' range shifts under climate change. Global Change Biology **10**, 2020-2027. doi: 10.1111/j.1365-2486.2004.00859.x

48. Killeen T.J., Douglas M., Consiglio T., Jørgensen P.M., Mejia J. (2007) Dry spots and wet spots in the Andean hotspot. Journal of Biogeography **34**, 1357-1373. doi: 10.1111/j.1365-2699.2006.01682.x

49. Guisan A., Tingley R., Baumgartner J.B., Naujokaitis-Lewis I., Sutcliffe P.R., Tulloch A.I.T., . . . Buckley Y.M. (2013) Predicting species distributions for conservation decisions. Ecology Letters **16**, 1424-1435. doi: 10.1111/ele.12189

50. Bombi P., Luiselli L., D'Amen M. (2011) When the method for mapping species matters: Defining priority areas for conservation of African freshwater turtles. Diversity and Distributions **17**, 581-592. doi: 10.1111/j.1472-4642.2011.00769.x

51. Peter B. W. J., and Possinghamm H. P. (2011) Optimizing Search Strategies for Invasive Pests: Learn before You Leap. Journal of Applied Ecology 48:1, 86–95. https://doi.org/10.1111/j.1365-2664.2010.01893.x.

52. Rodrigues A. S. L. et al. (2004). Effectiveness of the global protected area network in representing species diversity. Nature 428:640–643 edition. 10.1038/nature02422. Nature Publishing Group.

53. Vos A. D., Cumming G. S. (2019). The contribution of land tenure diversity to the spatial resilience of protected area networks. People and Nature 1:331–346 edition. https://doi.org/10.1002/pan3.29.

54. Bivand R. and Lewin-Koh N. (2018). maptools: Tools for Handling Spatial Objects. R package version 0.9-3. https://CRAN.R-project.org/package=maptools

55. Cantú-Salazar L, Gaston KJ. 2010. Very Large Protected Areas and Their Contribution to Terrestrial Biological Conservation. BioScience 60:808–818 edition. 10.1525/bio.2010.60.10.7.

56. Balmford A, Gaston KJ, Blyth S, James A, Kapos V. 2003. Global variation in terrestrial conservation costs, conservation benefits, and unmet conservation needs. Proceedings of the National Academy of Sciences 100:1046–1050 edition. 10.1073/pnas.0236945100. National Academy of Sciences.

57. Lessmann J, Fajardo J, Bonaccorso E, Bruner A. 2019. Cost-effective protection of biodiversity in the western Amazon. Biological Conservation 235:250–259 edition. 10.1016/j.biocon.2019.04.022.

58. Convention on Biological Diversity (2010) The Strategic Plan for Biodiversity 2011-2020 and the Aichi Biodiversity Targets (Decision X/2). Nagoya, Japan, https://www.cbd.int/doc/decisions/cop-10/cop-10-dec-02-en.pdf.

59. Dinerstein, Eric, David Olson, Anup Joshi, Carly Vynne, Neil D. Burgess, Eric Wikramanayake, Nathan Hahn, et al. "An Ecoregion-Based Approach to Protecting Half the Terrestrial Realm." BioScience 67, no. 6 (June 1, 2017): 534–45. https://doi.org/10.1093/biosci/bix014.
